# Mutation allele burden remains unchanged in chronic myelomonocytic leukaemia responding to hypomethylating agents

**DOI:** 10.1038/ncomms10767

**Published:** 2016-02-24

**Authors:** Jane Merlevede, Nathalie Droin, Tingting Qin, Kristen Meldi, Kenichi Yoshida, Margot Morabito, Emilie Chautard, Didier Auboeuf, Pierre Fenaux, Thorsten Braun, Raphael Itzykson, Stéphane de Botton, Bruno Quesnel, Thérèse Commes, Eric Jourdan, William Vainchenker, Olivier Bernard, Noemie Pata-Merci, Stéphanie Solier, Velimir Gayevskiy, Marcel E. Dinger, Mark J. Cowley, Dorothée Selimoglu-Buet, Vincent Meyer, François Artiguenave, Jean-François Deleuze, Claude Preudhomme, Michael R. Stratton, Ludmil B. Alexandrov, Eric Padron, Seishi Ogawa, Serge Koscielny, Maria Figueroa, Eric Solary

**Affiliations:** 1 INSERM U1170, Gustave Roussy, 114, rue Edouard Vaillant, 94805 Villejuif, France; 2 Department of Hematology, Gustave Roussy Cancer Center, 114, rue Edouard Vaillant, 94805 Villejuif, France; 3 INSERM US23, CNRS UMS3655, Gustave Roussy, 114, rue Edouard Vaillant, 94805 Villejuif, France; 4 Department of Pathology, University of Michigan Medical School, 1500 E Medical Center Dr, Ann Arbor, Michigan 48109, USA; 5 Department of Pathology and Tumour Biology, Kyoto University, Yoshida-Konoe-cho, Sakyo-ku, Kyoto 606-8501, Japan; 6 Université Lyon 1, UMR CNRS 5558, Université Claude Bernard, 16 rue Raphael Dubois, Lyon 69100, France; 7 Centre Léon Bérard, INSERM U1052, CNRS UMR5286, 8 Prom. Léa et Napoléon Bullukian, 69008 Lyon, France; 8 Department of Hematology, Assistance Publique–Hôpitaux de Paris, Hôpital Saint-Louis, 1 Avenue Claude Vellefaux, 75010 Paris, France; 9 Department of Hematology, Assistance Publique–Hôpitaux de Paris, Hôpital Avicenne, 125 Rue de Stalingrad, 93000 Bobigny, France; 10 Cancer Research Institute de Lille, INSERM U837, 1 Place de Verdun, 59000 Lille, France; 11 Institut de médecine régénératrice, Biothérapie et Institut de biologie computationnelle, INSERM U1040, Université de Montpellier, 80 avenue Augustin Fliche. 34295 Montpellier, France; 12 Department of Hematology, Centre Hospitalier Universitaire de Nîmes, Université Montpellier-Nîmes, 4 Rue du Professeur Robert Debré, 30029 Nîmes, France; 13 Laboratory of Genome Informatics, Kinghor Center for Clinical Genomics, Garvan Institute of Medical Research, 384 Victoria Street, Darlinghurst New South Wales 2010, Australia; 14 Centre National de Génotypage, 2 rue Gaston Crémieux CP 5721, 91 057 Evry, France; 15 Cancer Genome Project, Wellcome Trust Sanger Institute, Wellcome Trust Genome Campus, Hinxton, Cambridgeshire CB10 1SA, UK; 16 Theoretical Biology and Biophysics, Los Alamos National Laboratory, P.O. Box 1663, Los Alamos, New Mexico 87545, USA; 17 Center for Nonlinear Studies, Los Alamos National Laboratory, P.O. Box 1663, Los Alamos, New Mexico 87545, USA; 18 Department of Hematology, Malignant hematology, H. Lee Moffitt Cancer Center, 12902 USF Magnolia Drive, Tampa, Florida 33612, USA; 19 Department of Biostatistics, Gustave Roussy Cancer Center, 114 rue Edouard Vaillant, 94805 Villejuif, France; 20 Department of Hematology, Faculty of Medicine, University Paris-Sud, 63 Rue Gabriel Péri, 94270 Le Kremlin-Bicêtre, France

## Abstract

The cytidine analogues azacytidine and 5-aza-2'-deoxycytidine (decitabine) are commonly used to treat myelodysplastic syndromes, with or without a myeloproliferative component. It remains unclear whether the response to these hypomethylating agents results from a cytotoxic or an epigenetic effect. In this study, we address this question in chronic myelomonocytic leukaemia. We describe a comprehensive analysis of the mutational landscape of these tumours, combining whole-exome and whole-genome sequencing. We identify an average of 14±5 somatic mutations in coding sequences of sorted monocyte DNA and the signatures of three mutational processes. Serial sequencing demonstrates that the response to hypomethylating agents is associated with changes in DNA methylation and gene expression, without any decrease in the mutation allele burden, nor prevention of new genetic alteration occurence. Our findings indicate that cytosine analogues restore a balanced haematopoiesis without decreasing the size of the mutated clone, arguing for a predominantly epigenetic effect.

## Abstract (web summary)

Chronic myelomonocytic leukaemia is treated with agents that modify DNA methylation but whether they have direct cytotoxic effects is unclear. Here, the authors show that cells from treated patients show marked methylation changes without altered somatic mutation burden, suggesting that cytotoxicity is not a major factor in therapeutic efficacy.

## Graphical abstract

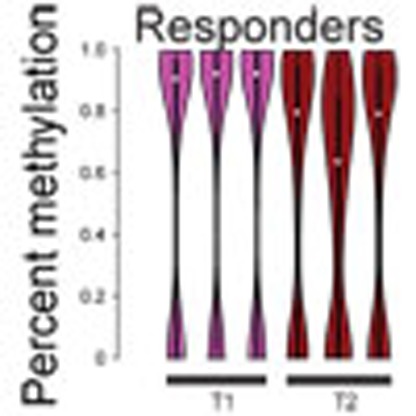

CMML, a clonal haematopoietic malignancy that usually occurs in the elderly, is the most frequent myelodysplastic syndrome/myeloproliferative neoplasm^1^. Nonspecific cytogenetic abnormalities are observed in 30–40% of cases^2^. More than 30 candidate genes were identified to be recurrently mutated in leukaemia cells^3^,^4^,^5^,^6^,^7^,^8^,^9^,^10^,^11^,^12^,^13^. Analysis of these recurrently mutated genes at the single cell level in 28 CMML bone marrow samples identified the main features of the leukaemic clone architecture, including the accumulation of mutations in the stem cell compartment with early clonal dominance, a low number of subclones, and a strong advantage to the most mutated cells with differentiation^4^. As in several other myeloid malignancies, *ASXL1* gene mutations demonstrated the strongest independent negative prognostic impact^14^,^15^.

The median overall survival of CMML patients is about 30 months, one-third evolving to acute myeloid leukaemia (AML) while the others die from the consequences of cytopenias. Allogeneic stem cell transplantation, which is the only curative therapy, is rarely feasible because of age. In patients ineligible for transplantation, intensive chemotherapy results in low response rates and short response duration^2^. The cytidine analogues azacytidine (AZA) and decitabine (5-aza-2′-deoxycytidine) were approved for the treatment of CMML^16^. These azanucleosides were originally described as cytotoxic drugs, but low doses also cause DNA demethylation by inactivation of DNA methyltransferases^17^,^18^. It remains unclear whether the response to these drugs, which is always transient, results from a cytotoxic or an epigenetic effect.

In this study, to tackle this issue, we completed a comprehensive analysis of genetic alterations in CMML cells by combining whole-exome (WES) and whole-genome sequencing (WGS). Then, we performed sequential WES and RNA sequencing (RNA-Seq) together with DNA methylation analyses in untreated patients and patients treated with a hypomethylating drug. Clinical response to cytidine analogues was associated with a dramatic decrease in DNA methylation, which was not observed when the disease remained stable on therapy. In responding patients, the size of the mutated clone remained unchanged, arguing for a predominantly epigenetic effect of these drugs.

## Results

### Genetic alterations in coding regions

**Figure 1:**
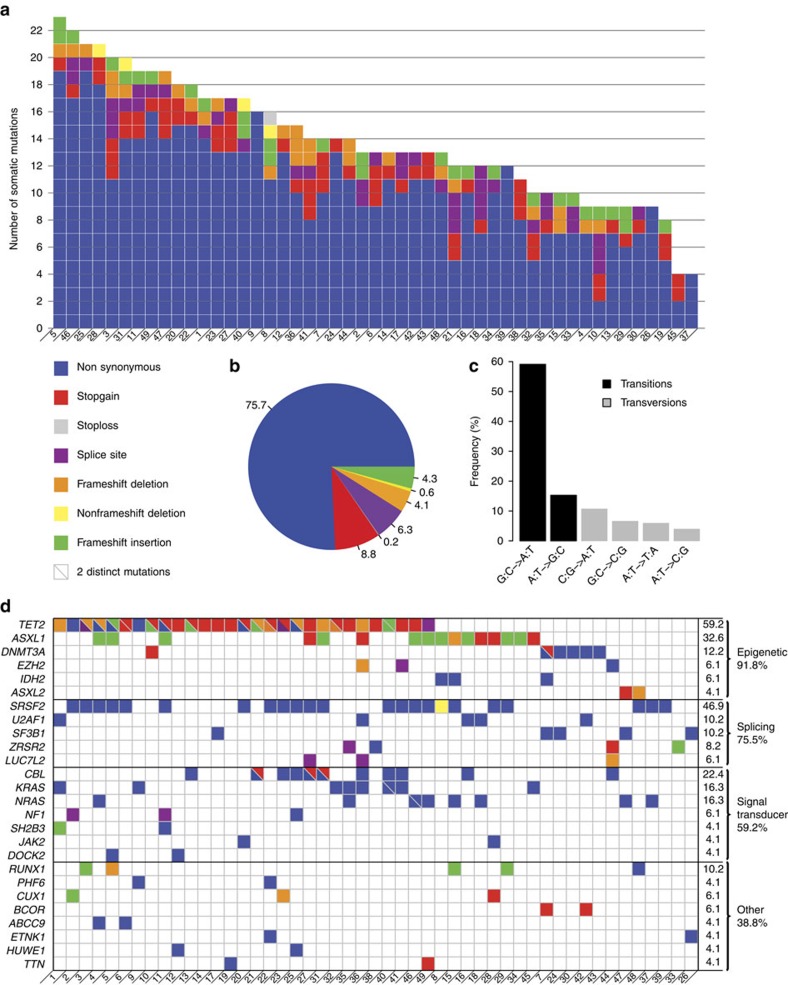
Somatic variants in coding regions identified by whole-exome sequencing. WES was performed in 49 chronic myelomonocytic leukaemia samples. (**a**) Number and type of somatic mutations identified in each patient designated as UPN, showing a majority of nonsynonymous variants. (**b**) Repartition of the 680 validated somatic variants identified in the 49 patients. (**c**) Repartition of base changes with transitions in black and transversions in grey. (**d**) Of the 36 recurrently mutated genes identified by WES, 26 are actively transcribed in CD14^+^ cells and CD34^+^ cells (according to Gene Expression Omnibus at http://www.ncbi.nlm.nih.gov/geo/). These 26 recurrently mutated genes are classified according to their function, including epigenetic regulation, pre-messenger RNA splicing, and signal transduction. Colours indicate the type of mutation. Two colours separated by a slash indicate two distinct mutations in the same gene.

Since it remained uncertain whether the most frequent recurrent gene mutations had been all identified, we performed WES of paired tumour–control DNA from 49 CMML cases (Supplementary Figs 1 and 2, Supplementary Tables 1 and 2). and validated 680 somatic mutations in 515 genes by deep resequencing (Supplementary Data 1). The average number of somatic mutations was 14±5 per patient (range: 4–23; Fig. 1a). The most frequent alterations were somatic nonsynonymous single-nucleotide variants (SNVs; *N*=515; 75.7%; Fig. 1b). Most of the 618 variants were transitions (*N*=453, 73.3%; Fig. 1c). We detected mutations affecting an epigenetic regulator gene in 45 out of 49 (91.8%) patients, a splicing machinery gene in 37 (75.5%) and a signal transduction gene in 28 (59.2%). Among the 36 genes found mutated in at least 2 patients, 19 had been previously identified in the context of CMML, validating previous screens of mutations in candidate genes in this specific disease^3^,^4^,^5^,^6^,^7^,^8^,^9^,^10^,^11^,^12^,^13^. *TET2*, *SRSF2* and *ASXL1* were confirmed to be the most frequently mutated genes in CMML^14^.

Of the 17 other recurrently mutated genes, only 7 were actively transcribed in CD14-positive^19^ and CD34-positive haematopoietic cells (according to Gene Expression Omnibus at http://www.ncbi.nlm.nih.gov/geo/). These genes include *ABCC9* (ATP-binding cassette, sub-family C member 9), *ASXL2* (additional sex combs-like 2), *DOCK2* (dedicator of cytokinesis protein 2), *HUWE1* (HECT, UBA and WWE domain containing 1, E3 ubiquitin protein ligase), *NF1* (Neurofibromin 1), *PHF6* (PHD finger protein 6) and *TTN* (Titin). Altogether, recurrent mutations were identified in 26 genes expressed in haematopoietic cells (Fig. 1d). Constitutive truncating mutations in *TTN* gene were recently validated as a cause of dilated cardiomyopathy^20^ and the variants identified in CMML samples were validated by an independent method. Except this very large gene, the whole coding sequence of the 6 other genes, whose recurrent mutation in the context of CMML had not been described previously, was deep sequenced in an additional cohort of 180 patients (Supplementary Table 3). Of the 229 studied patients, the most frequently mutated gene was *PHF6* (*N*=17; 7.4%). *NF1* was altered in 14 (6.1%) patients. *DOCK2* and *ABCC9* mutations were detected, respectively, in five samples (2.1%), *HUWE1* mutations in three samples (1.3%) and *ASXL2* mutations in two samples (Supplementary Table 4). On average, each patient had 3.1 alterations (range: 1–7) among the 26 recurrently mutated genes identified in this series. Combinations are summarized in Supplementary Fig. 3 and relationships with clinical and biological features in Supplementary Table 5.

**Figure 2:**
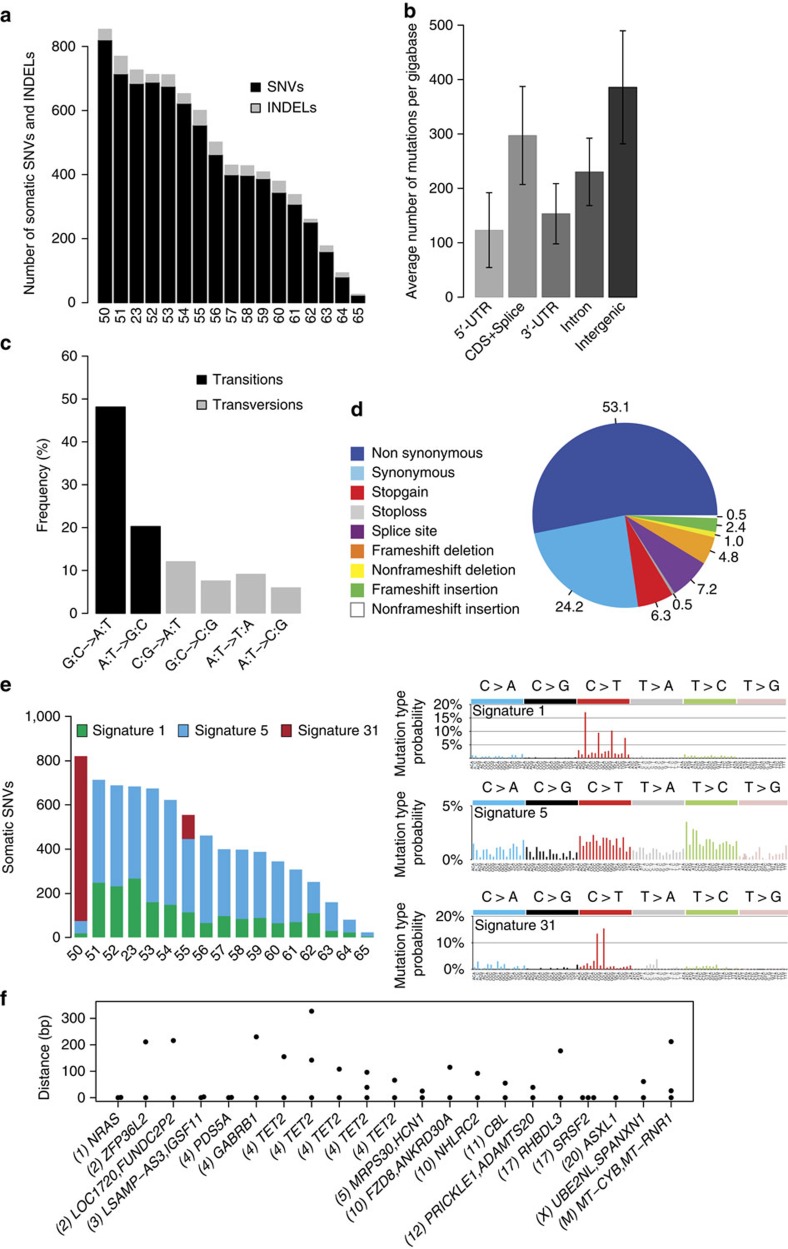
Somatic variants in coding and non-coding regions identified by whole-genome sequencing. WGS was performed in 17 chronic myelomonocytic leukaemia samples (including one analysed by WES). (**a**) Number of somatic single-nucleotide variants and short insertions/deletions in each patient. (**b**) Repartition of the 8077 somatic variants, expressed as numbers of variants per gigabase, identified across the genomic regions. Mean and 95% confidence intervals (*n*=17) are shown. (**c**) Repartition of base changes with transitions in black and transversions in grey. (**d**) Repartition of the 207 somatic variants identified in coding regions. (**e**) Mutational signatures extracted from whole genomic analyses. (**f**) Potential hotspots of mutations (two variants less than 250 bp apart) including nine in coding regions of driver genes (including *TET2*, *ASXL1*, *SRSF2*, *CBL* and *NRAS*), two in intronic regions of *PDS5A* and *NHLRC2*, one in 3'UTR of *ZFP36L2*, six in intergenic regions and 1 in the mitochondrial chromosome. Numbers between comas indicate the chromosome number.

We extended this analysis by performing WGS of paired tumour–control DNA from 17 patients. Of the 8,077 somatic variants identified (Fig. 2a, Supplementary Table 6 and Supplementary Data 2), 207 were located in coding regions or splice sites (11.8 per patient; Fig. 2b) and the combination of WES and WGS identified two additional recurrently mutated genes that are actively transcribed in haematopoietic cells, ten-eleven translocation 3 (*TET3)* and proline-rich coiled-coil 2B (*PRRC2B*). All these additional recurrent abnormalities may contribute to CMML phenotype heterogeneity.

### *TET3* loss of function mutation

**Figure 3:**
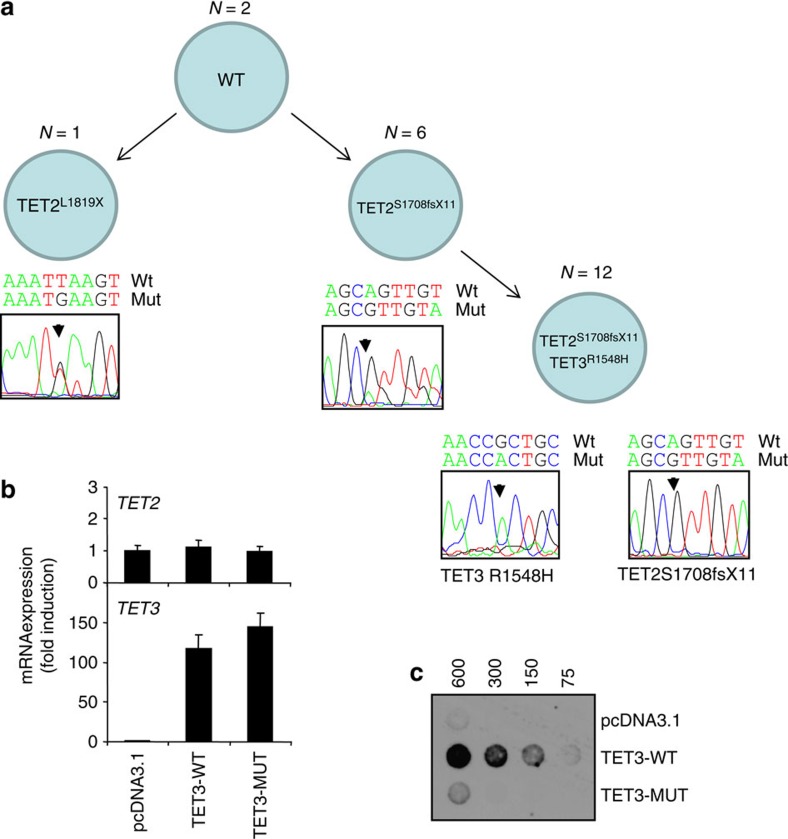
TET3–R1548H mutation inhibits 5hmC modification. (**a**) Single cell analysis of TET3R1548H, TET2S1708fsX11 and TET2L1819X mutations in sorted CD14^+^ cells from UPN22. (**b**) *TET2* and *TET3* gene expression measured by quantitative reverse transcriptase–PCR in HEK293T cells transfected with the pcDNA3.1 empty vector or pcDNA3.1 encoding wild-type (TET3-WT) and R1548H TET3 (TET3-MUT). Reporter gene: *RPL32*. Results are related to pcDNA3.1 control. Error bars represent mean±standard deviation of triplicates. (**c**) Dot blot analysis of 5-hydroxymethylcytosine (5hmC) on genomic DNA (4-fold serial dilutions in ng) isolated from HEK293T cells transfected as in **b**.

*TET3* mutations are very infrequent in haematologic diseases^21^,^22^ and were not detected in myeloid malignancies so far^23^. In the two patients with a mutated TET3 gene, the two alleles of TET2 were also mutated. We further explored the functional consequences of TET3^R148H^ identified in UPN22. Genetic analyses of CD14^+^ cells at the single cell level (N=21) identified a complex repartition of TET2 and TET3 mutations, with TET2^S1708fs^ being either alone or in combination with TET3^R148H^, whereas TET2^L1819X^ was detected in only one TET3 wild-type cell (Fig. 3a). Expression of wild-type and TET3^R148H^ alleles in HEK293T cells (Fig. 3b) demonstrated that TET3^R148H^ mutation impaired the enzyme ability to promote 5-methylcytosine hydroxylation (Fig. 3c). Since many functional redundancies have been identified between TET2 and TET3 dioxygenases (for review see ref. 24), future studies are necessary to elucidate a potential cooperative interaction between TET2 and TET3 mutated alleles in diseased cells.

### Genetic alterations in non-coding regions

Further analysis of WGS data indicated that, on average, CMML cells carried 475 (range: 27–854) somatic variants in their DNA (Fig. 2a), 6.3% being short insertions and deletions. These variants were mostly in intergenic (63.5%) and intronic (31.5%) regions (Fig. 2b). Somatic SNVs (93.7%) were mostly transitions (66.3%; Fig. 2c), and synonymous base changes represented 24.1% of the identified variants (Fig. 2d). Our computational framework for extracting mutational signatures^25^ identified the signatures of three mutational processes (Fig. 2e). Two (signatures 1 and 5) were previously observed^26^ and believed to be due to clock-like mutational processes operative in normal somatic tissues. Interestingly, we identified in two cases a novel mutational signature (signature 31) characterized by C:G>T:A mutations at CpCpC and CpCpT (mutated based underlined) and exhibiting a strong transcriptional strand bias (Supplementary Fig. 4). We did not detect any recurrent alteration in non-coding regions, as described in other tumour types^27^,^28^,^29^. We identified 21 potential hotspot regions with at least 2 variants in distinct samples being at most 250 bp far (Fig. 2f). Nine were in the coding sequence of recurrently mutated genes, and 3 in non-coding regions of genes transcribed in haematopoietic cells (*PDS5A*, *ZFP36L2* and *NHLRC2*). Finally, we detected 147 variants in promoters and 37 variants in permissive enhancers, of which 3 showed activity in blood cells (Supplementary Data 3)^30^.

### Serial whole-exome analyses

**Figure 4:**
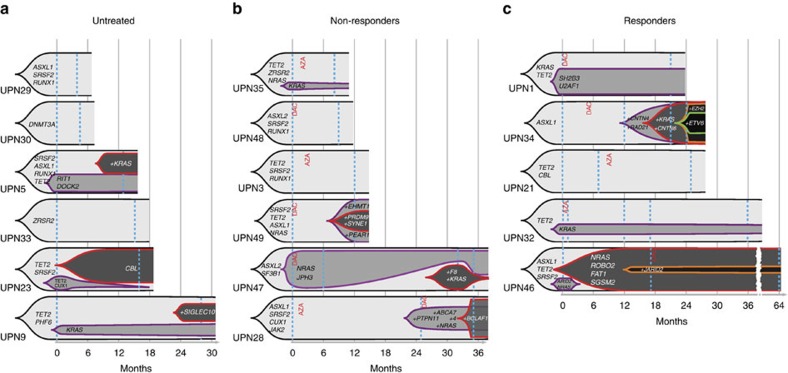
Serial whole-exome sequencing analysis of somatic variants. WES of sorted peripheral blood monocyte DNA was performed two- to fivefold in 17 patients at a mean interval of 14±8 months (range: 4–32). The clonal evolution of recurrently mutated genes is shown. UPN indicates the patient number. A selection of the variants detected by the first whole-exome sequencing is shown (all the variants identified in each individual patient are depicted in Supplementary Fig. 5). All the changes in variant allele frequency and new variants detected by repeating whole-exome sequencing are shown. Black indicates the founding clone and subsequent subclones are shown in violet, red, orange, and green, successively. Patients were either untreated (**a**) or treated with either azacytidine (AZA) or decitabine (DAC) as indicated in red. Blue dash lines indicate WES. (**b**) Patients with a stable disease on therapy. (**c**) Responding patients.

WES of sorted monocyte DNA was repeated in 17 patients. The mean time between two analyses was 14±8 months (range: 4–32). Six patients received supportive care, whereas 11 were treated with either AZA (*N*=5) or decitabine (*N*=6). The number of serial analyses per patient ranged from two to five (Supplementary Fig. 1 and Table 7). The mean duration of treatment was 21±13 months (range: 5–47). One or two WES were performed before treatment, subsequent analyses being performed on therapy in samples collected immediately before the next cycle. Five of the treated patients demonstrated a response at the time of sampling (‘responders'), including one complete response (UPN32), three marrow complete responses with haematological improvement and one marrow complete response without haematological improvement (UPN34). In the six other patients, the disease remained stable on therapy, without haematological improvement (‘non-responders')^18^,^31^. In total, we performed 27 serial WES analyses. In 17 cases, we did not detect any change in gene mutations as compared with the previous analysis, the mutated allele burden remaining stable in all patients but two (UPN23 and UPN47; Fig. 4). In responding patients, hypomethylating agents did not decrease the mutated allele burden in circulating monocytes. In eight cases, we detected changes in the number of mutated genes, including three untreated, three non-responders with a stable disease and one responder (Fig. 4 and Supplementary Fig. 5). The latter was a 74-year-old man (UPN34) with 12 somatic mutations at diagnosis who successively acquired mutations in *CNTN4* and *RAD21* genes, then in *KRAS*, *CNTN6* and *PCDHGA6* genes while being in complete marrow response without haematological improvement. The last exome analysis, performed in acute transformation, identified an *EZH2/ETV6* mutated subclone (Supplementary Fig. 5). UPN46 was analysed first while being untreated, showing the disappearance of a subclone with *ARID2* and *NRAS* mutations while another clone with *NRAS*, *ROBO2*, *FAT1* and *SGSM2* mutations expanded. This patient was subsequently treated with decitabine and responded to treatment, without change in mutation number and allele burden (Fig. 4c, Supplementary Fig. 5 and Fig. 6).

**Figure 5:**
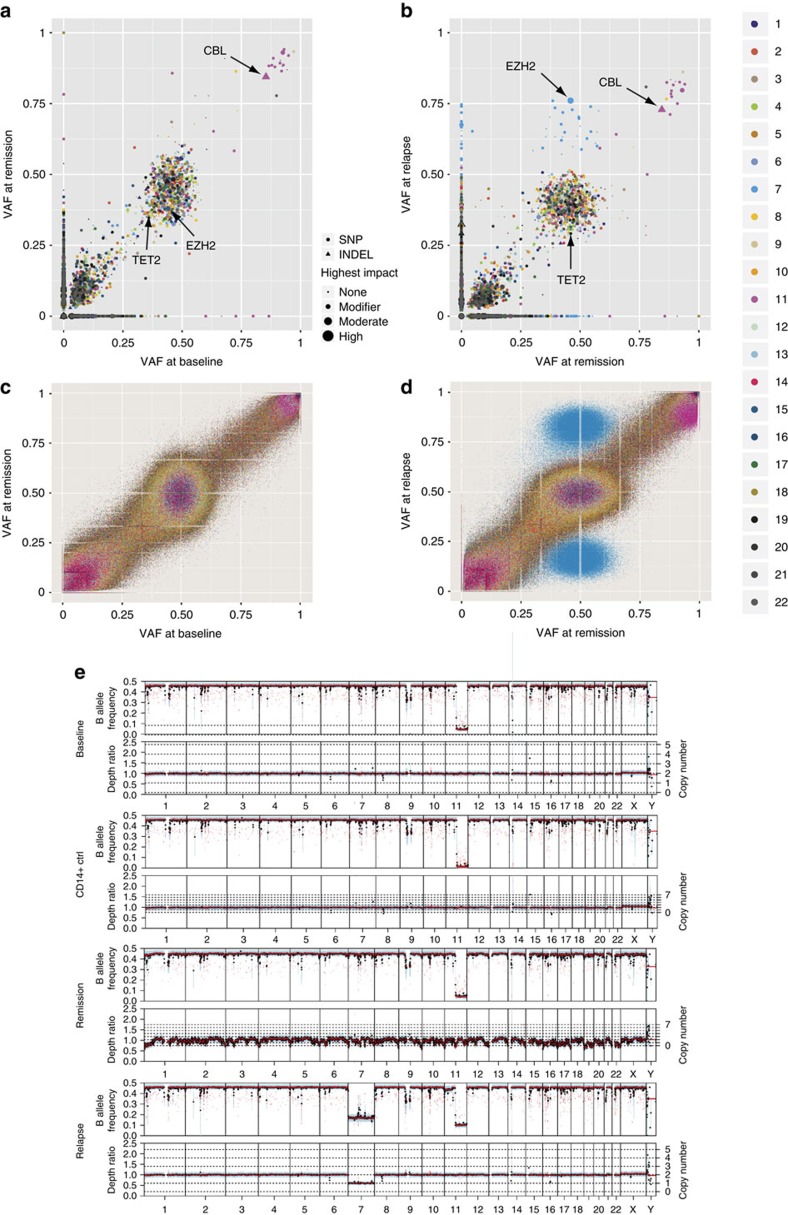
Serial whole-genome sequencing in a 5-AZA exceptional responder. WGS was performed before 5-azatidine treatment (baseline), in complete response (remission) and at disease progression (relapse). (**a**,**b**) Scatter plot of somatic variants identified at baseline, remission, and progression. Chromosomal location is color coded and the size of the object denotes its predicted impact on protein function. High impact variants are those that are predicted to have the highest likelihood of altering protein expression or function such as frameshifts or nonsense variants. Circles denote single-nucleotide variants and triangles denote insertions or deletions. (**c**,**d**) Scatter plot of all variants identified with Freebayes at baseline, remission, and progression. Chromosomal location is color coded. (**e**) Copy number changes as identified from whole-genome sequencing data using Sequenza.

In one additional patient who demonstrated a long and complete response to AZA, then progressed to AML (Methods section), serial WGS of bone marrow mononucleated cells^32^ was performed. Before AZA therapy, somatic variants in *TET2*, *EZH2* and *CBL* genes were identified. In a best response sample, a striking stability of variant allele frequency was observed. At the time of progression, a loss of heterozygocity of mutated *EZH2* was detected, together with the acquisition of a mutation in *ASXL1*, and a whole loss of chromosome 7, which was confirmed by serial cytogenetic analysis (Fig. 5). This observation emphasizes the lack of genetic response to AZA and the possibility to detect genetic progression on therapy, preceding progression to acute leukaemia.

### Gene expression and DNA methylation

**Figure 6:**
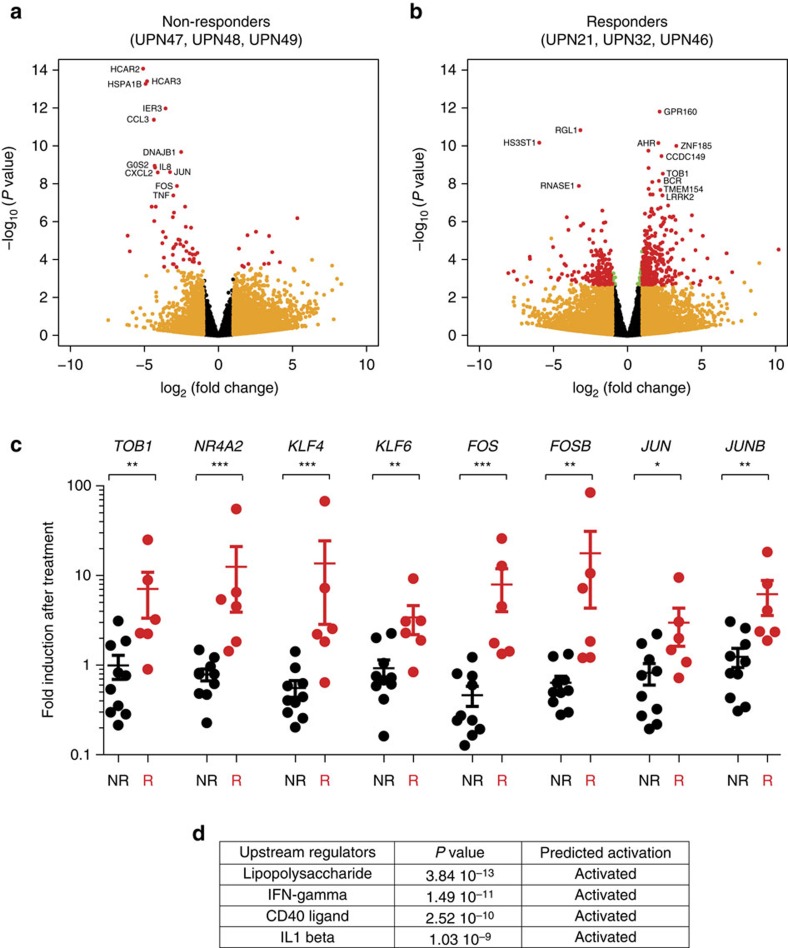
Evolution of gene expression pattern on hypomethylating agent therapy. Gene expression was analysed at two time points in sorted peripheral blood monocytes from 9 chronic myelomonocytic leukaemia patients, including three untreated and six treated with either azacytidine or decitabine. These cases were randomly selected in each group. Three treated patients remained stable on therapy (non-responders) whereas the three others were responders. In treated patients, the first sample was collected before treatment, the second one after at least 5 drug cycles and just before the next cycle. Volcano plots of genes differentially expressed between these two time points are shown in non-responders (**a**) and in responders (**b**). The name of the most differentially deregulated genes is indicated. No significant change in gene expression was detected in untreated patients analysed twice at an at least 5-month interval (see also Table 1). Each dot (*N*=24,563) represents a gene; green dots, padj ≤0.05, orange dots, abs (log_2_ (fold change)) ≥1 and red dots, padj ≤0.05 and abs(log_2_ (fold change)) ≥1. (**c**) Quantitative reverse transcriptase–PCR validation of the differential expression of 8 genes in 6 responders (3 studied by RNA sequencing in **b** and 3 additional cases) and 10 non-responders (3 studied by RNA sequencing in **a** and 7 additional cases). Normalizer gene, *RPL32*. Similar results were obtained with two other normalizer genes, *GUS* and *HPRT* (Supplementary Fig. 8). (**d**) Significant changes in pathways detected by analysing RNA sequencing data with Ingenuity (www.ingenuity.com/products/ipa).

In nine of these patients, we performed serial RNA-Seq (Fig. 6, Supplementary Tables 8 and 9), the first sample being collected before treatment. Three remained untreated, and six were treated with a hypomethylating drug, the second sample being collected on therapy. Of the six treated patients, three were responders, the three others remaining on therapy with stable disease (non-responders). We measured the effect of time on gene expression. We noticed a strong impact of treatment in responders, with 513 differentially expressed genes, whereas only 63 genes were differentially expressed in treated patients with stable disease (non-responders), and none in untreated patients (Table 1, Fig. 6a,b and Supplementary Data 4). The proportions of significantly differentially expressed genes between the groups were all significantly different (*P*<10^−10^, *χ*^2^-test). Quantitative reverse transcription–PCR analysis validated all the tested upregulated genes in an extended cohort of 6 responders compared with 10 patients with stable disease (Fig. 6c and Supplementary Fig. 7 and Fig. 8).

**Figure 7:**
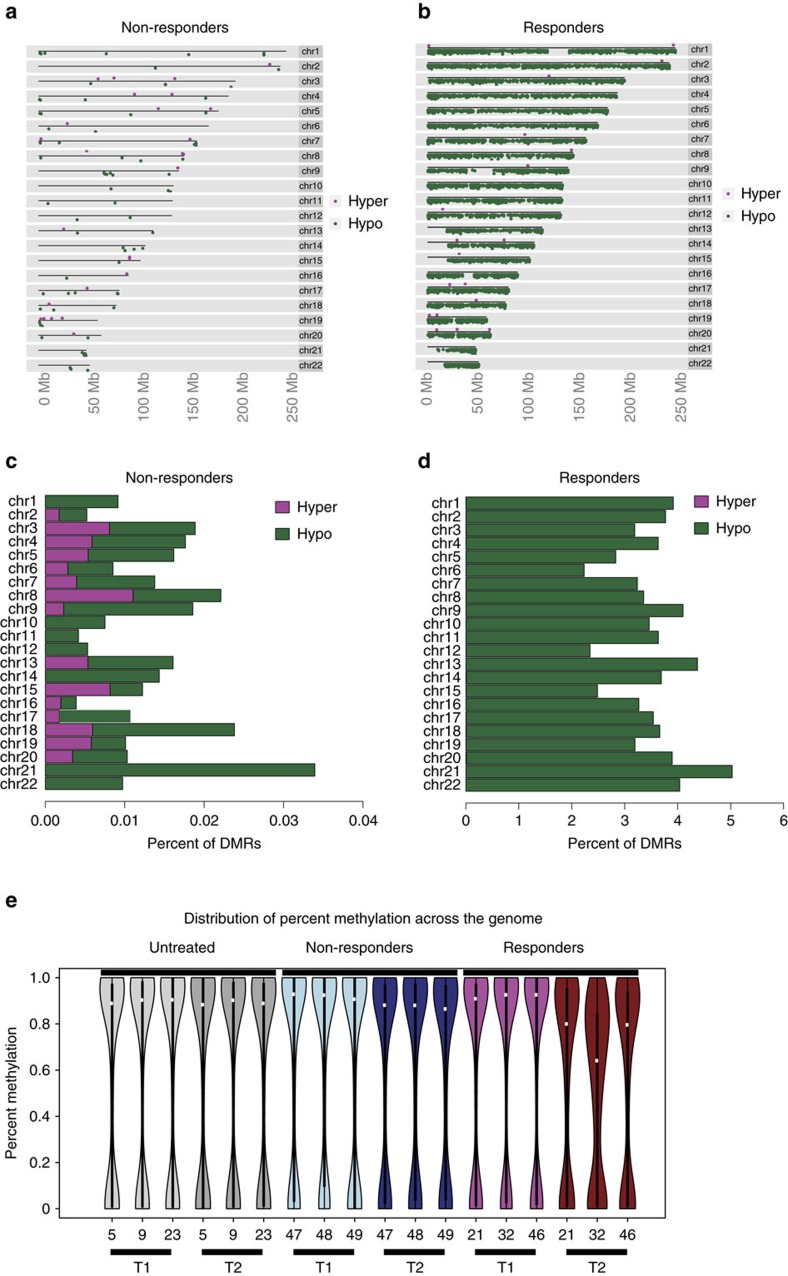
Evolution of DNA methylation pattern on hypomethylating drug therapy. Methylation was analysed at two time points in sorted monocytes from nine chronic myelomonocytic leukaemia patients, including three untreated and six treated with either azacytidine or decitabine. Three treated patients remained stable on therapy (non-responders) whereas the three others were responders. In treated patients, the first sample was collected before treatment, the second one after at least five drug cycles and just before the next cycle. (**a**,**b**) Chromosome ideograms representing differentially methylated regions (DMRs) in non-responders (**a**) and in responders (**b**) are shown. Reduction in DNA methylation is in green, whereas increased methylation is in pink. (**c**,**d**) Barplots showing the percentage of genomic regions with significant changes in DNA methylation in non-responders (**c**) and in responders (**d**) are also shown. No change was identified in the 3 untreated patients (Table 1). (**e**) Violin plots showing the evolution of global methylation change in each patient (untreated patients in grey, treated with a stable disease (non-responders) in blue, treated responders in red with the lighter color indicating the earliest analysis.

**Figure 8:**
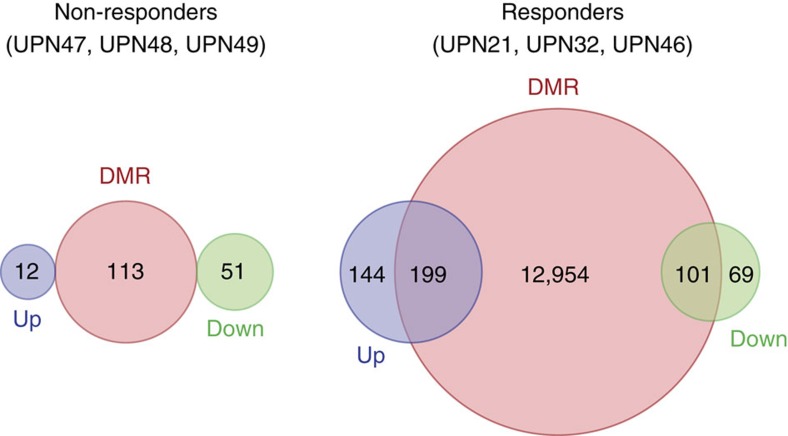
Relationship between changes in DNA methylation and in gene expression. Venn diagrams of interactions between differentially methylated regions (DMR, red circles) and differentially expressed genes (upregulated in blue; downregulated in green) as defined in Fig. 5 (padj ≤0.05 and abs(log_2_ (fold change)) ≥1).

Finally, we explored the effect of time on methylation status in the same samples by using the enhanced reduced-representation bisulfite sequencing assay (Fig. 7). Differentially methylated regions (DMRs) between the two time points were defined by a more than 25% change in methylation and a false discovery rate (FDR) ≤10%. Differential methylation was detected almost exclusively in the three responding patients (Fig. 7b,d,e). The number of DMRs remained low in non-responding patients with a stable disease under therapy (Fig. 7a,c,e) and no change was identified in untreated patients (Table 1, Supplementary Fig. 9 and Supplementary Data 5). Changes observed in responding patients were predominantly demethylation, whereas changes detected in treated patients with a stable disease included both gains and losses of DNA methylation (Supplementary Fig. 9). In responders, DMRs were significantly depleted in promoters and in CpG islands while being enriched in generic enhancers (Supplementary Fig. 10). Some overlap was detected between DMRs and changes in gene expression in responders, which was not observed in non-responders (Fig. 8).

## Discussion

This first comprehensive analysis of genetic alterations in CMML cells demonstrates that azanucleosides, although inducing dramatic changes in DNA methylation and gene expression in responding patients, do not reduce the mutated allele burden, nor permit the re-expansion of wild-type haematopoietic cells.

Previous screening of candidate genes identified somatic mutations in *TET2*, *ASXL1* and *SRSF2* genes as the most frequent recurrent events in CMML cells^4^. Our comprehensive analysis validates this molecular fingerprint and identifies additional recurrent abnormalities that may contribute to the disease phenotype heterogeneity. Several of the most recurrent mutations identified in leukaemic cells were associated with age-related clonal haematopoiesis^33^,^34^,^35^ or ‘silent' pre-leukaemic clones^36^,^37^,^38^. The bias in myeloid differentiation towards the granulomonocytic lineage that characterizes CMML could be related to the expansion of such a clone, for example, due to early clonal dominance of *TET2* (refs 4, 39). In this setting, the occurrence of an additional mutation resulting in a stringent arrest of differentiation leads to acute-phase disease^38^,^40^, as illustrated by sequential analyses in UPN34 who partially responded to decitabine for 2 years until the emergence of an *EZH2*/*ETV6* mutated subclone and an acute leukaemia phenotype. Importantly, this observation indicates that the response to a hypomethylating agent does not prevent the accumulation of genetic damage in the leukaemic clone.

The number of genetic alterations identified in the genome of CMML cells was close to that observed in other haematological malignancies^26^. Most somatic variants identified were transitions, with a predominance of C:G->T:A, and a mutational signature suggesting that the historical mutational process was related mostly to ageing^26^. Accordingly, the number of variants identified in juvenile CMML, another myeloproliferative neoplasm/myelodysplastic disease that occurs in young children, is much lower than that measured in CMML^41^.

Although these results do not exclude some cytotoxic effect of azanucleosides, their epigenetic activity appears to play a central role in restoring a more balanced haematopoiesis in the 30–40% of CMML patients who respond to these drugs^17^,^18^. Immunophenotyping analyses already suggested that these drugs could eliminate bulk blast cells without eradicating leukaemia stem and progenitor cells in AML patients^42^ and did not correct CD34^+^ cell immunophenotypic aberrancies in CMML patients^43^. Mutations in epigenetic genes observed in almost every CMML case lead to DNA hypermethylation^44^ and epigenetically controlled changes in gene expression contribute to the disease phenotype, as demonstrated for transcription intermediary factor-1γ (*TIF1γ*) gene whose epigenetic downregulation was identified in a fraction of patients, and whose deletion in the myeloid compartment induces a CMML phenotype in the mouse^45^. Clinical response to hypomethylating drugs is associated with a re-expression of this gene when initially downregulated^45^, indicating that hypomethylating drugs can suppress epigenetic changes that contribute to the disease phenotype. This epigenetic effect could decrease the competitiveness of the most mutated cells in the progenitor and stem cell compartment^4^,^40^ but not the mutated allele burden in the mature cell compartment. Also, although we have shown before that the number of subclones in the immature compartment was usually low in CMML patients, we cannot rule out that an impact of treatment on clonal architecture in the bone marrow participates to the generation of a more balanced haematopoiesis.

Clinical trials have shown that 30–40% of CMML patients respond to azanucleosides^2^,^18^. Since epigenetic changes were observed only in responders, specific patterns of epigenetic changes may be amenable to reversion by azanucleosides^17^. We have shown that differentially methylated non-promoter regions of DNA at baseline distinguished responders from non-responders to decitabine^46^, whereas the pattern of somatic mutations did not^18^. Some epigenetic patterns could also prevent the activity of hypomethylating drugs by either decreasing the expression of human nucleoside transporters and metabolic enzymes needed for their activation such as cytidine and deoxycytidine kinases and cytidine deaminase^16^,^47^ or increasing the expression of genes encoding cytokines such as CXCL4 and CXCL7 that, when released, could antagonize the drug effects^46^. In two responding patients, prolonged administration of azanucleosides, although improving haematopoiesis, did not prevent the accumulation of genetic events, ultimately leading to acute transformation, indicating that these drugs do not prevent genetic evolution of the leukaemic clone. Further analyses are needed to determine whether they could even promote such genetic evolution.

The present findings have clinical implications. First, prolonged administration of hypomethylating drugs may not have any benefit in CMML patients when haematological improvement is not observed after a few cycles. Second, these drugs could increase the survival of responding patients by restoring a more balanced haematopoiesis, but they might not prevent the occurrence of new genetic events leading to acute transformation. Finally, better analysis of how these drugs modulate the immunogenicity of mutated cells could lead to combination of hypomethylating agents with immune checkpoint blockers as nucleoside analogues render the cells more immunogenic through inducing the expression of cancer testis antigens^48^, promoting the demethylation of programmed death-1 immune checkpoint molecule^49^, and inducing retrovirus activation^50^,^51^, suggesting that an interaction of epigenetic drugs and immunotherapeutic approaches^52^ might be considered. Our results also raise the question on whether epigenetic targeting molecules currently developed to treat haematological malignancies^53^,^54^ will eradicate mutated cells or erase the epigenetic consequences of these mutations, leading to the transient restoration of a more balanced haematopoiesis.

## Methods

### Patients

Peripheral blood and bone marrow samples were collected on ethylenediaminetetraacetic acid from 245 patients with a CMML diagnosis according to the World Health Organisation criteria^1^. When indicated, several peripheral blood samples were collected sequentially from a given patient (Supplementary Fig. 1). We initially performed WES in 49, WGS in 17 and validation of recurrent mutations by deep sequencing in 180 cases. Serial WES were performed in 17 patients, including 6 untreated and 11 treated with either decitabine (*N*=6; EudraCT 2008-000470-21 GFM trial; NCT01098084; https://www.clinicaltrials.gov/)^18^ or AZA (*N*=5; following the European Medicines Agency approval; EMEA/H/C/000978). Responses were classified according to the International Working Group 2006 criteria^31^. Patients with stable disease without haematological improvement remained treated until progression^17^. When indicated, sequential RNA-Seq and DNA methylation analysis^46^ were performed. In treated patients, samples were collected immediately before the following drug cycle. All the procedures were approved by the institutional board of Gustave Roussy and the ethical committee Ile de France 1, and written informed consent was obtained from each patient. Data collected from French and Japanese patients were analysed homogeneously. Patient characteristics are in Supplementary Table 1, the flow chart of analyses in Supplementary Fig. 1.

### Cell sorting

Bone marrow (*N*=9) or peripheral blood (*N*=7) mononucleated cells were separated on Fycoll-Hypaque. Peripheral blood CD14^+^ monocytes were sorted with magnetic beads and the AutoMacs system (Miltenyi Biotech, Bergish Gladbach, Germany)^45^. Control samples were peripheral blood CD3-positive T lymphocytes sorted with the AutoMacs system or buccal mucosa cells (*N*=3) or skin fibroblasts (*N*=12). All the samples used in the validation cohort (*N*=180) were sorted peripheral blood CD14^+^ monocytes. DNA and RNA were extracted from cell samples using commercial kits. Monocytes were sorted for DNA sequencing on the basis of our previous analysis of CMML clonal architecture showing the growth advantage to the most mutated cells^4^, and flow cytometry analysis of peripheral blood monocytes showing limited phenotypic alteration in the classical monocyte population in patients treated with hypomethylating drugs, even though responders have more intermediate and non-classical monocytes^19^. In one patient, bone marrow mononucleated cells were used for serial WGS. *TET2* and *TET3* gene sequencing in UPN22 were performed in single CD14^+^ cells sorted using C1 (Fluidigm) after whole-genomic DNA amplification.

### Functional analysis of mutated *TET3*

pcDNA3.1-*TET3R1548H* was generated using Q5 site-directed mutageneis (New England Biolabs Evry, France) before transfecting HEK293T cells with constructs encoding wild-type or mutated *TET3*. After 2 days in culture, DNA was extracted and 5-hydroxymethylcytosine was detected as previously described^39^.

### Whole-exome sequencing

We performed WES in 49 patients at diagnosis. In 17 of them, 2–5 serial analyses were done. 1 μg of genomic DNA was sheared with the Covaris S2 system (LGC Genomics, Molsheim, France). DNA fragments were end-repaired, extended with an ‘A' base on the 3′-end, ligated with paired-end adaptors and amplified (six cycles) using a Bravo automated platform (Agilent technologies). Exome-containing adaptor-ligated libraries were hybridized for 24 h with biotinylated oligo RNA baits, and enriched with streptavidin-conjugated magnetic beads using SureSelect (Agilent technologies, Les Ulis, France). The final libraries were indexed, pooled and paired-ends (2 × 100 bp) sequenced on Illumina HiSeq 2000 (San Diego, CA). In nine cases, WES was performed in Japan following a previously described protocol^55^. The mean coverage in the targeted regions was 112 × (Supplementary Table 2). Two individual cases have been reported in our previous studies^4^,^8^. Sequencing data are deposited at the European Genome–Phenome Archive (EGA), hosted by the European Bioinformatics Institute (EBI), under the accession number EGAS00001001264.

### WES analysis

Raw reads were aligned to the reference human genome hg19 (Genome Reference Consortium GRCh37) using BWA 0.5.9 (Burrows–Wheeler Aligner) backtrack algorithm with default parameters. PCR duplicates were removed with Picard (http://picard.sourceforge.net) version 1.76. Local realignment around indels and base quality score recalibration were performed using GATK 2.0.39 (Genome Analysis ToolKit). Statistics on alignment and coverage are given in Supplementary Table 2. SNVs and indels were called with VarScan2 somatic 2.3.2 (ref. 56). Reads and bases with a Phred-based quality score ≤20 were ignored. Variants with somatic *P* value below 10^−4^ (or 10^−3^ for samples with mean coverage <100 × or contamination >15% in CD3^+^ control sample) were reported. In addition to the Fisher's exact test of VarScan, we required (variant allele frequency in the tumour sample–variant allele frequency in the normal sample) ≥15% to distinguish somatic from germline variations. Variants were annotated with Annovar. Mutations were searched in 1000G (April 2012) and Exome Sequencing Project (ESP5400). Conservation of the position was predicted by PhyloP and the effect of the mutation was predicted by SIFT, Polyphen2, LRT and MutationTaster. We excluded variants reported in dbSNP version 129, filtered variants located in intergenic, intronic, untranslated regions and non-coding RNA regions, and removed synonymous SNVs and variants with mapping ambiguities. A mutation was reported as present if variant allele frequency (VAF) ≥4%.

### Targeted deep sequencing

Regarding exome validation, Ion AmpliSeq Custom Panel Primer Pools were used to perform multiplex PCR for preparation of amplicon libraries. Briefly, 20 ng of DNA per primer pool quantified using a Qubit Fluorometer (Invitrogen, Carlsbad, CA) were used in the multiplex PCR. Unique indexed libraries per sample were generated, quantified by Qubit, pooled and run on an Ion 318 Chip using the Ion PGM Sequencer (Life Technologies). Seventy one per cent of the candidates for somatic mutation were confirmed by deep resequencing at a mean coverage of 759 ×. In total, we validated 680 somatic mutations (Supplementary Table 3). Also, the whole coding regions of genes found mutated in at least two patients and expressed in myeloid cells were deep sequenced (mean coverage, 690 ×) in a cohort of 180 CMML patients (Supplementary Table 4 and Table 5). Ion AmpliSeq Custom Panel Primer Pools were used (10 ng of genomic DNA per primer pool) to perform multiplex PCR. Libraries were generated with addition of paired-end adaptors (NEXTflex, Bioo Scientific) before paired-end sequencing (2 × 150 bp reads) using an Illumina MiSeq flow cell and the onboard cluster method (Illumina, San Diego, CA). Quality of reads was evaluated using FastQC (http://www.bioinformatics.bbsrc.ac.uk/projects/fastqc/). Raw reads were filtered with Trimommatic 0.30 (ref. 57) to remove adaptors, truncate any read whose average quality on a sliding window (six bases) was ≤20, remove the start and the end of a read if ≤20 and any read with an average quality ≤20 or a length <36. Statistics on alignment and coverage are given in Supplementary Table 2 and detailed analysis of each studied variant in Supplementary Table 3. Targeted resequencing was analysed similarly to WES except the suppression of PCR duplicates. We added the following public databases: ESP 6500, dbSNP 138, COSMIC 68 (Catalogue Of Somatic Mutations In Cancer) and ClinVar (20140303).

### Prediction of driver genes

We applied DrGaP (driver genes and pathways)^58^ to synonymous and nonsynonymous somatic variants (889 in 694 genes) to measure the probability of each variant to occur by chance. Among the 22 genes with FDR ≤10%, 20 were mutated in at least 2 patients and actively transcribed in myeloid cells (*MIER* and *FIBIN* genes carried 2 variants in a unique patient, respectively). Six of the 26 recurrently mutated and actively transcribed genes were mutated in only 2 patients: *SH2B3* (FDR=0.22), *PHF6* (FDR=0.22), *DOCK2* (FDR=0.30), *ABCC9* (FDR=0.36), *HUWE1* (FDR=0.78) and *TTN* (FDR=0.88).

### Whole-genome sequencing

We performed WGS in 17 patients at diagnosis, including one already studied by WES. Genomic DNA (1 μg) was sheared to 300–600 bp (average size=398±14 bp) using a Covaris E210 (Covaris, Woburn, Massachusetts, USA). Libraries for 101 bp paired-end sequencing were prepared according to the Truseq PCR free protocol (Illumina). Library quality was evaluated by quantitative PCR for quantification (Kapa Biosystems Ltd., London, UK) and by low output sequencing on Miseq (Illumina) for clusterisation efficiency. Samples were loaded on HiSeq 2000 and sequenced. Quality of reads was evaluated using FastQC. Sequences were filtered with Trimommatic. Reads were aligned to the reference genome hg19 using BWA MEM algorithm 0.7.5a with default parameters. The PCR duplicates were removed with Picard 1.94. Local realignment and base quality score recalibration were performed using GATK 2.7.4. The mean coverage of all the samples was 31 ×. Detailed statistics on alignment and coverage are given in Supplementary Table 6. Somatic SNVs were identified by SomaticSniper 1.0.3 (ref. 59), VarScan2 2.3.7 and Strelka 1.0.14 (ref. 60). We conserved somatic variants with ≥15 × in normal, ≥6 × in tumour and ≥3 reads supporting the variant. We used a SomaticScore Tumour ≥30 for SomaticSniper, a Somatic *P* value ≤0.01 for VarScan2 and a QSS_N/QSI_NT≥15 for Strelka. We ran Strelka with the following parameters: ssnvNoise=0.000000005, sindelNoise=0.00000001, ssnvPrior=0.001, sindelPrior=0.001 and extraStrelkaArguments: -used-allele-count-min-qscore 20 and min-qscore 20. The other parameters were set by default. We removed SNVs annotated as SpanDel, BCNoise or DP in FILTER field. We excluded INDELs reported as OVERLAP or defined as Repeat, iHpol, BCNoise or DP in the FILTER field. In addition, we required (variant allele frequency in the tumour sample–variant allele frequency in the normal sample) ≥20% and excluded the variants whose allele frequency in the normal sample was ≥15% to differentiate somatic from germline mutations. We removed the variants located in low complexity regions, immunoglobulin loci (as reported in http://www.genecards.org/ for *TCRA*, *TCRB*, *TCRG*, *IGH*, *IGL* and *IGK*) and genes in which false positives have been frequently detected by new-generation sequencing^61^. By removing low complexity regions, as defined in the masked genome chromOut.tar.gz generated by repeatMasker (http://hgdownload.soe.ucsc.edu/goldenPath/hg19/bigZips/), we removed 45% of the genome, thereby eliminating 74, 69 and 71% of the SNVs detected by using SomaticSniper, VarScan2 and Strelka, respectively, and 87 and 77% of the indels identified by VarScan2 and Strelka, respectively. In subsequent analyses, the SNVs and indels identified by combining these stringent algorithms were used. Sequencing data are deposited at the EGA hosted by the EBI under the accession number EGAS00001001264.

### Potential hotspots in promoters and enhancers

First, sequential windows were used to calculate the probability for a 250-bp region to carry at least two variants in two distinct patients among 17 patients. The probability to find at least 2 mutations in one of the 6.82 × 10^6^ windows of 250 bp defined in non-repeated regions of the genome among 17 patients was 10^−3^. Second, we defined a potential hotspot region as a region in which, in a sequence shorter than 250 bp, two variants were identified in at least two patients. With that method, from the 8,077 variants detected (Supplementary Data 2), we identified 21 clusters of variants on a same chromosome at most 250 bp far, defining potential hotspots (Supplementary Data 3). We detected 147 variants in 144 distinct promoter regions (from −2,000 bp before to +200 bp after the translation starting site obtained from UCSC website on 07 November 2014) and 37 variants in the 43,011 enhancer regions reported in ref. 30, of which three were located in the 3,795 enhancers whose activity is ≥5% in blood and ≥5% in monocytes.

### Independent case report from Lee Moffitt Cancer Center

A 57-year-old female patient progressed ∼10 months after diagnosis of a type-1 CMML according to the World Health Organization definition with normal cytogenetics, prompting the initiation of 5-azacitidine therapy. After four cycles of therapy, the patient had a complete remission that persisted for 30 cycles. Disease progression was suspected because of a declining platelet count and confirmed by an increase in bone marrow myeloblasts. 5-azacitidine was discontinued and the patient transformed to AML 8 months later. Bone marrow mononucleated cells^32^ were collected before the treatment start, during complete response and at progression. The following part of the study was approved by the H. Lee Moffitt Cancer Center institutional review boards and the patient provided informed consent before initiating sequencing procedures under the Total Cancer Care protocol. WGS was performed on five lanes for each leukaemia sample, and two lanes for the CD3+ germline on the Illumina HiSeq X platform. The goal was to achieve 125 and 60 × depth, respectively. Sequencing data was aligned to b37d5 reference genome with BWA MEM, and duplicates were marked, and multiple lanes merged using novosort. Somatic SNV and INDEL variant calling was performed using Strelka for tumour normal pairs. Somatic copy number variants, loss of heterozygosity regions, ploidy and purity were determined using Sequenza. Freebayes with minimum VAF=0.01 was used to generate variants from individual samples, and to assess the number of clones. Variants were annotated using Variant Effect Predictor. Phylosub was used to reconstruct the evolutionary lineage of samples, using either high, or medium- and high-impact variants (loss of function vs missense, respectively).

### RNA sequencing

Sequential RNA-Seq was performed on 18 samples (9 patients) with high-quality RNA (RNA Integrity Score ≥7.0 as determined by the Agilent 2100 Bioanalyzer). RNA was quantified using a Qubit Fluorometer (Invitrogen, Cergy-Pontoise, France). RNA-Seq libraries were prepared using the SureSelect Automated Strand Specific RNA Library Preparation Kit as per manufacturer's instructions (Agilent technologies) and a Bravo automated platform (Houston, TX). Briefly, 150 ng of total RNA sample was used for poly-A mRNA selection using oligo(dT) beads and subjected to thermal mRNA fragmentation. The fragmented mRNA samples were subjected to complementary DNA synthesis and further converted into double stranded DNA that was used for library preparation. The final libraries were bar-coded, purified, pooled together in equal concentrations and subjected to paired-end (101 bp) sequencing on HiSeq2000 (San Diego, CA). Two separate samples were multiplexed into each lane. Quality of reads was evaluated using FastQC.

### RNA-Seq analysis

Sequences were filtered with Trimommatic and alignment was performed with Tophat2 version 2.0.9 (ref. 62) and Bowtie2 version 2.1.0 (ref. 63). The filtered reads were aligned to a reference transcriptome (downloaded from UCSC website on 20 December 2013). The remaining reads were split and segments were aligned on the reference genome, as described^62^. In average, 88.95% of reads were aligned (Supplementary Table 9) and counted with HTSeq (v0.5.4p5) (ref. 64) using the following parameters: --mode=intersection-nonempty --minaqual=20 --stranded=no. Differential expression analysis was performed using DESeq2 package version 1.6.3 (ref. 65) with R statistical software version 3.1.2. To study the effect of time in each of the three groups (Supplementary Data 4), we used a generalized linear model to explain the counting Y_i_: Y_i_∼Group:Patient+Time+Group+Group:Time where Group indicates the status (untreated, responders and stable disease). We used independent filtering to set aside genes that have no or little chance to be detected as differentially expressed. To test the effect of time in each group, we used three contrasts defined as linear combinations of factor level means. Validation of RNA-Seq data was performed by quantitative PCR analysis in a selection of eight genes, using three independent genes as reporters (Supplementary Fig. 8).

### Genome-wide DNA methylation by ERRBS

Twenty-five nanograms of high-molecular weight genomic DNA were used to perform the ERRBS assay as previously described^66^ and sequenced on a HiSeq2000 Illumina sequencer. 50 bp reads were aligned against a bisulfite-converted human genome (hg19) using Bowtie and Bismark^67^. Downstream analysis was performed using R version 3.0.3, Bioconductor 2.13 and the MethylSig 0.1.3 package. Only genomic regions with coverage between 10 and 500 × were used for the downstream analysis (Supplementary Data 5). DMR were identified by first summarizing the methylation status of genomic regions into 25-bp tiles and then identifying regions with absolute methylation difference ≥25% and FDR <10%. DMRs were annotated to the RefSeq genes using the following criteria: (i) DMRs overlapping with a gene were annotated to that gene, (ii) intergenic DMRs were annotated to all neighbouring genes within a 50-kb window, and (iii) if no gene was detected within a 50-kb window, then the DMR was annotated to the nearest TSS.

## Additional information

**Accession codes:** Whole-exome sequencing, whole-genome sequencing, RNA sequencing and eRRBS raw sequence files (fastq files) have been deposited in the European Genome-phenome Archive (EGA), under accession code https://www.ebi.ac.uk/ega/studies/EGAS00001001264.

**How to cite this article:** Merlevede, J. *et al*. Mutation allele burden remains unchanged in chronic myelomonocytic leukaemia responding to hypomethylating agents. *Nat. Commun.* 7:10767 doi: 10.1038/ncomms10767 (2016).

## Supplementary Material

Supplementary Information Supplementary Figures 1-10 and Supplementary Tables 1-9.

Supplementary Data 1 List of the 680 somatic mutations validated by re-sequencing

Supplementary Data 2 Somatic variants (N=8077) detected by whole genome sequencing

Supplementary Data 3 Somatic variants identified in hotspot (N=46), promoter (N=147) and enhancer (N=37) regions of the genome.

Supplementary Data 4 Effect of time on gene expression. List of differentially expressed genes (abs(log2FoldChange)>=1)

Supplementary Data 5 Effect of time on genome methylation.

## Figures and Tables

**Table 1:** Changes induced by hypomethylating agents in gene expression and DNA methylation.

	Untreated	Treated non-responders	Treated responders
Number of patients	3	3	3
Time between analyses (months) mean±s.d.	17±10	9±3	27±17
			
*Changes in gene expression*			
Up	0	12	343
Down	0	51	170
Total	0	63	513
			
*Differentially methylated regions*			
Up	0	28	19
Down	1	75	35,895
Total	0	103	35,914

Genomic analyses were performed at two time points in sorted peripheral blood monocytes of nine chronic myelomonocytic leukaemia patients, including three left untreated and six patients treated with either azacytidine or decitabine. Among treated patients, 3 had a stable disease under therapy (non-responders) and three demonstrated clinical response (Figs 4 and 5). The first sample was collected before treatment, the second after at least five cycles of either azacytidine or decitabine, just before the next cycle. We measured the number of differentially expressed genes having abs(log_2_ (fold change)) ≥1 between T1 and T2, and the number of differentially methylated regions having ≥25% difference between T1 and T2.
