# Supplementary material for: Mutation allele burden remains unchanged in chronic myelomonocytic leukaemia responding to hypomethylating agents
Source: Nat Commun. 2016 Feb 24;7:10767. doi: 10.1038/ncomms10767 (PMC4770084; doi:10.1038/ncomms10767)
Supplement: Supplementary Information — Supplementary Figures 1-10 and Supplementary Tables 1-9. [file ncomms10767-s1.pdf]

Supplementary Figure 1 - Flowchart of the experiments

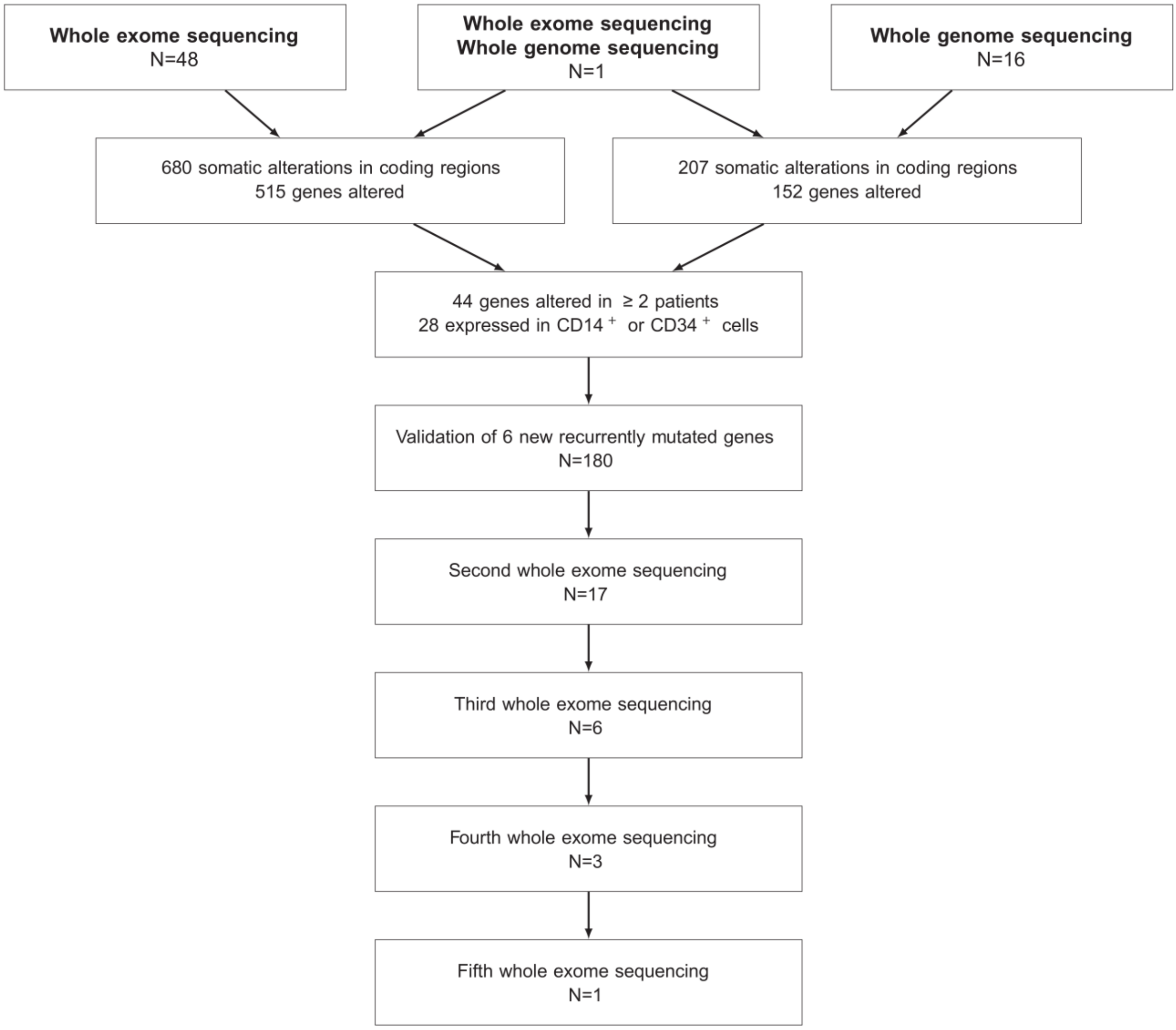

Supplementary Figure 2: Comparison of skin fibroblasts and CD3+ cells as germline controls. In UPN 23, we used CD3+ and skin fibroblasts as controls of whole exome and whole genome sequencing of CD14+ sorted monocyte DNA, respectively. In both analyses, the same abnormalities were detected in the coding regions of monocyte DNA. More variation in variant allele frequencies was observed in whole genome sequences, due to lower coverage.

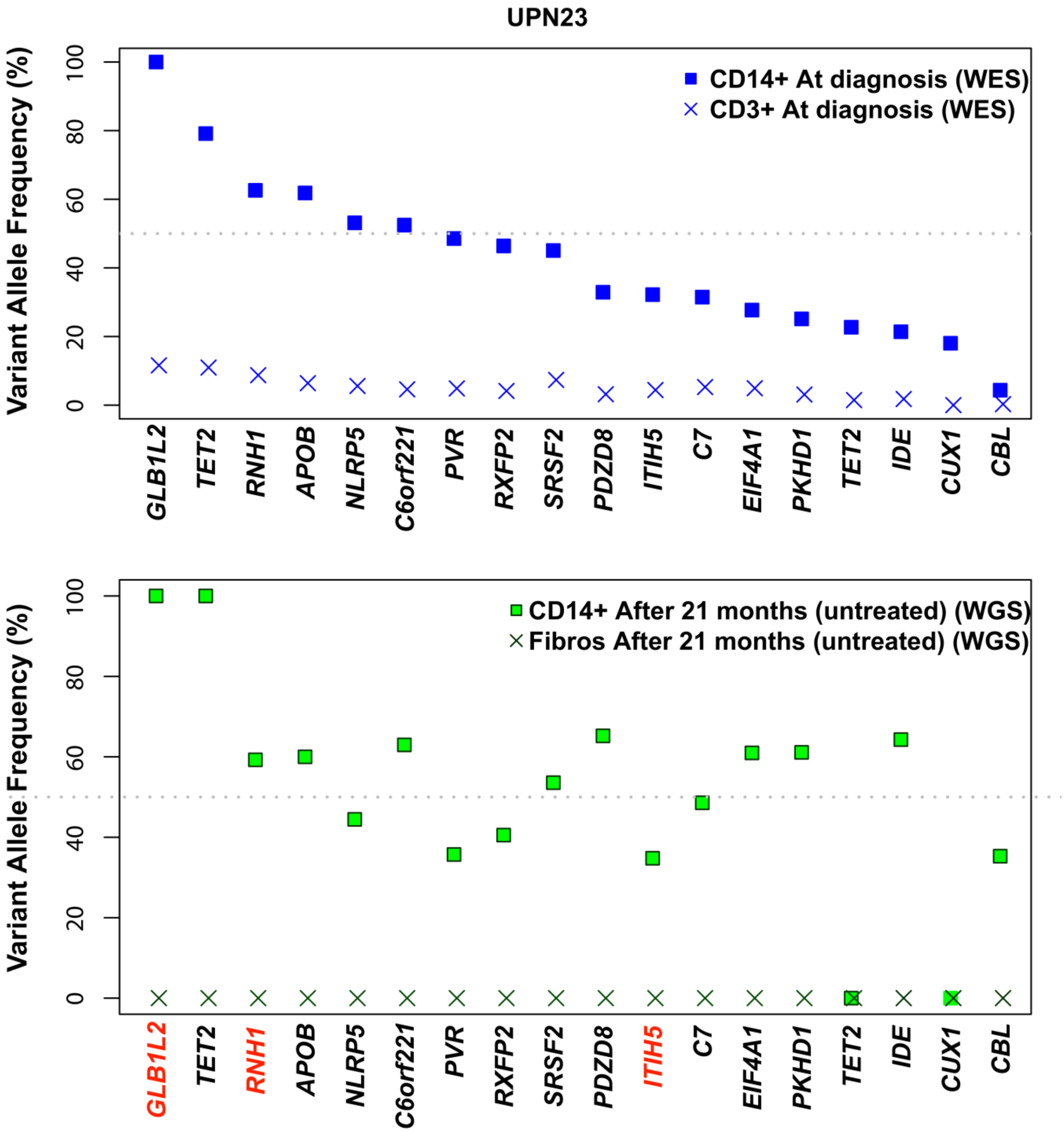

Supplementary Figure 3: Positive and negative correlations between recurrent gene mutations. P value as by Spearman test.

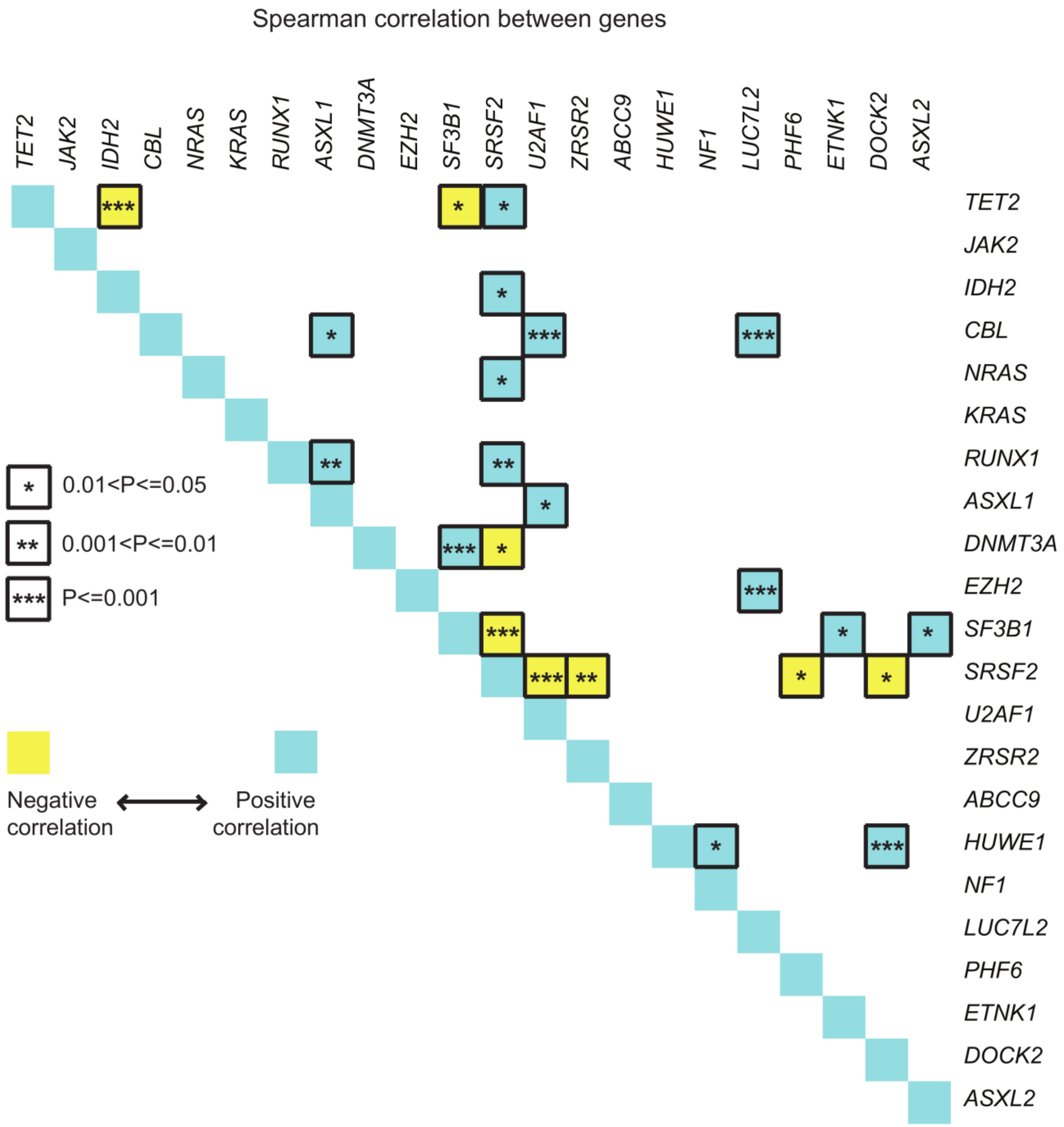

Supplementary Figure 4: Characteristics of mutational signature 31. The new signature 31 is characterized by C:G>T:A mutations at CpCpC and CpCpT (mutated based underlined) and exhibits a strong transcriptional strand bias (especially in regards to C:G>T:A mutations, with mutations occurring predominately on guanine) as illustrated below.

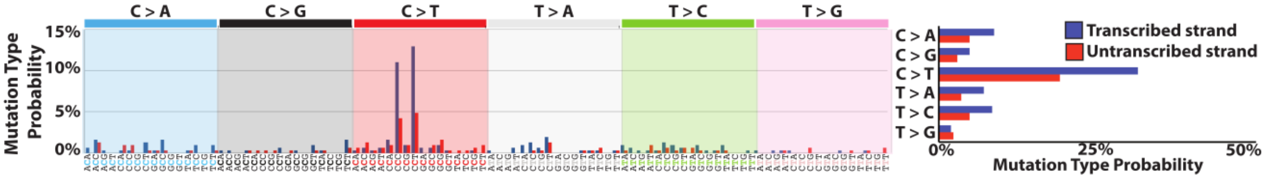

Supplementary figure 5: Allele frequency of the variants detected by serial whole exome sequencing of sorted CD14+ monocyte DNA in 17 patients. We did not detect any change in the number of variants by serial analyses shown on supplementary figures 5 to 8 and 10 to 15, including 4 untreated patients (29,30,33,23), 3 non responding patients with a stable disease upon treatment with a demethylating agent (35,48,3) and 3 patients who responded to treatment with a demethylating agent (32,1,21). We observed changes in the number of variants by serial analyses shown on supplementary figures 9, 16 to 21, including 3 untreated patients [5, 9 and 46 (46 being treated afterwards)], 3 so-called "non-responding" patients with a stable disease upon treatment with a demethylating agent (47,49,28) and one patient who responded to the demethylating drug (34).

1. Serial whole exome sequencing in patient 29

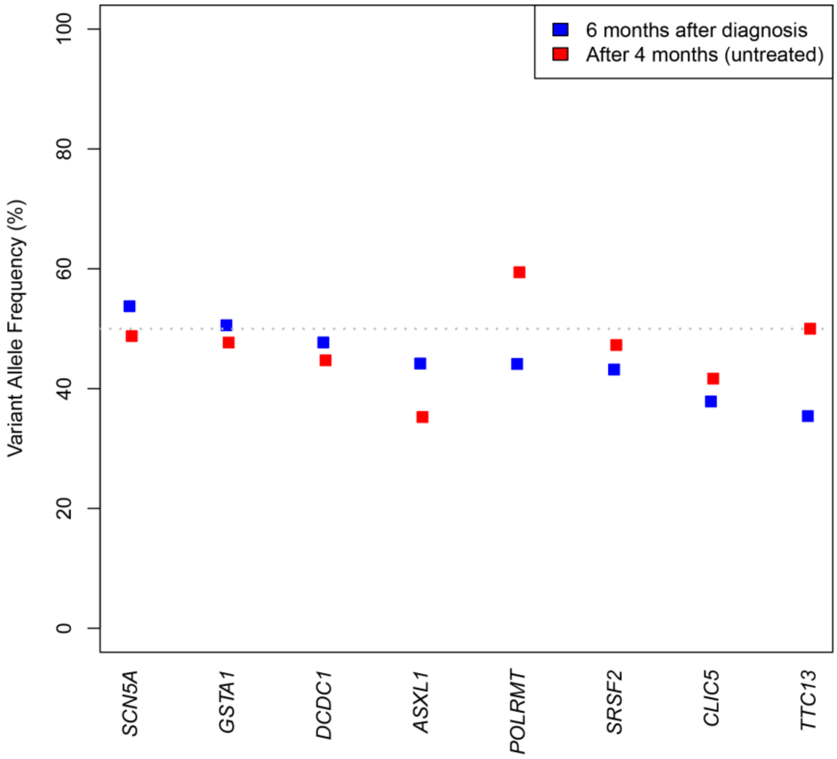

2. Serial whole exome sequencing in patient 30

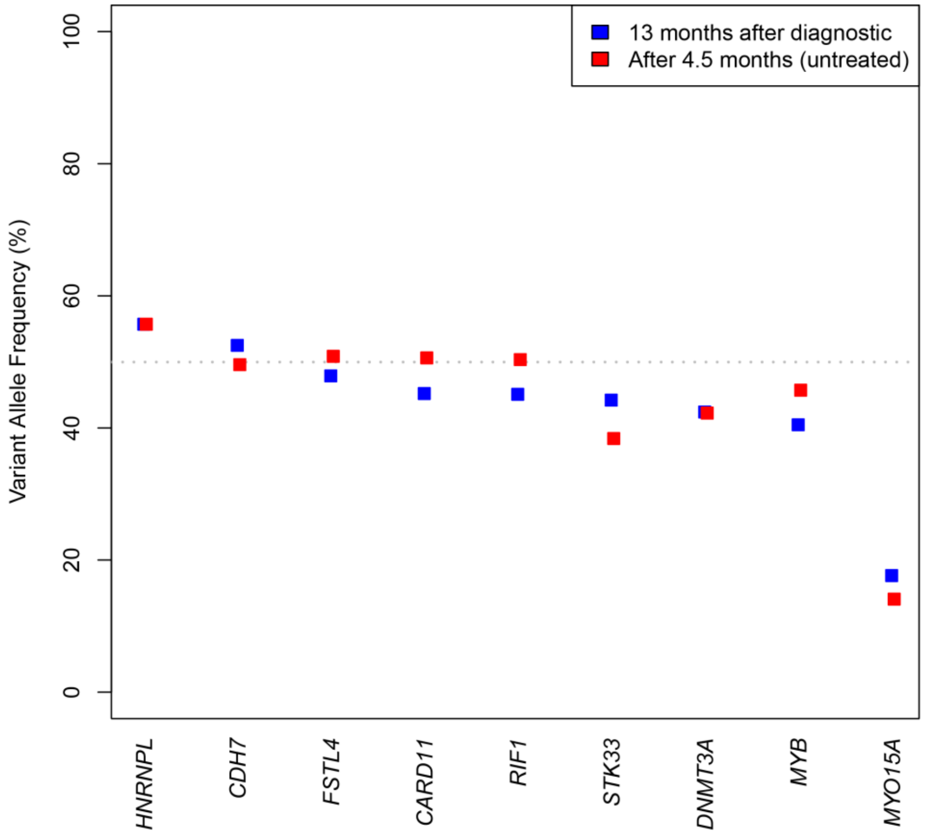

3. Serial whole exome sequencing in patient 33

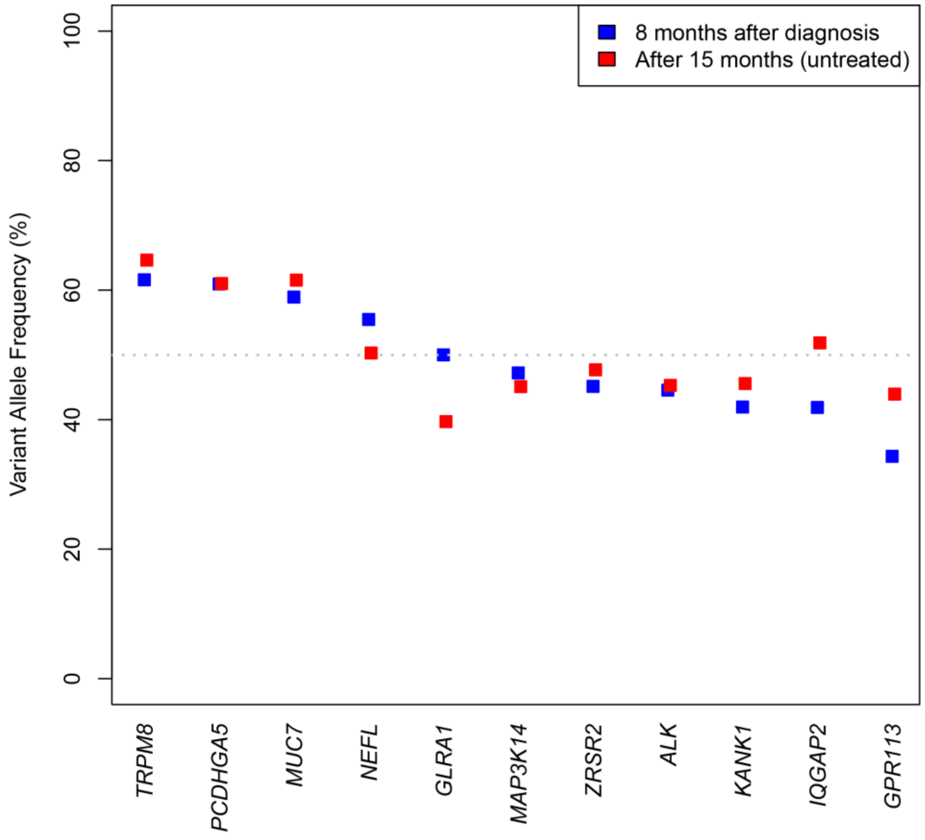

4. Serial whole exome sequencing in patient 23

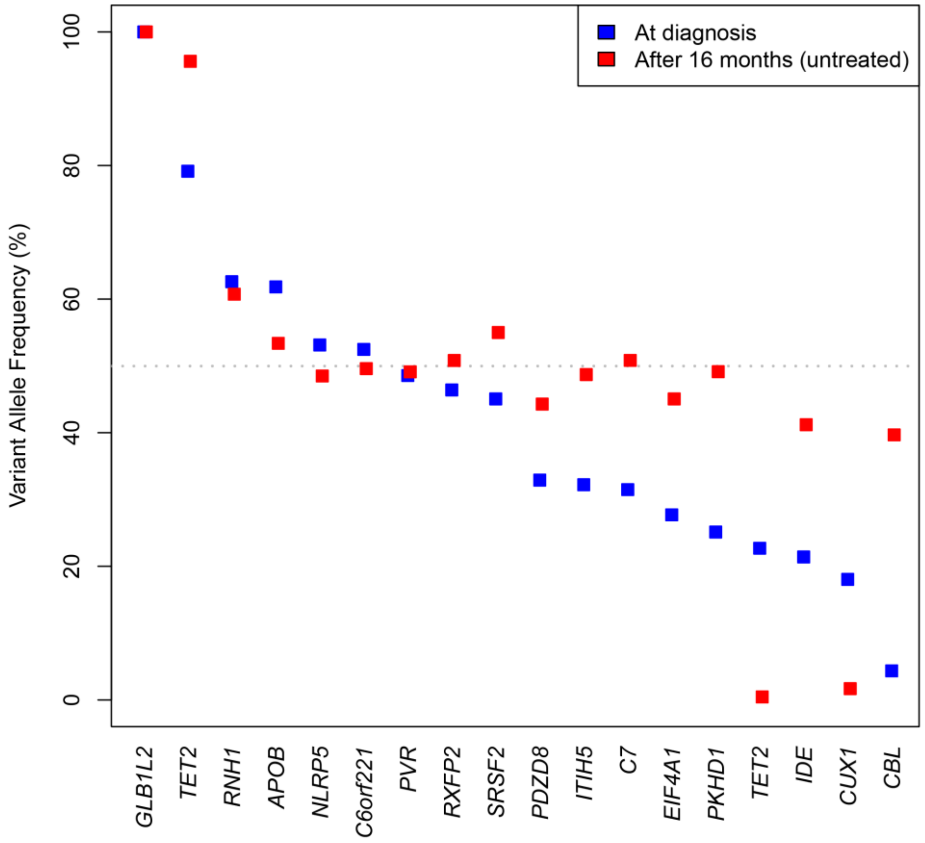

5. Serial whole exome sequencing in patient 47

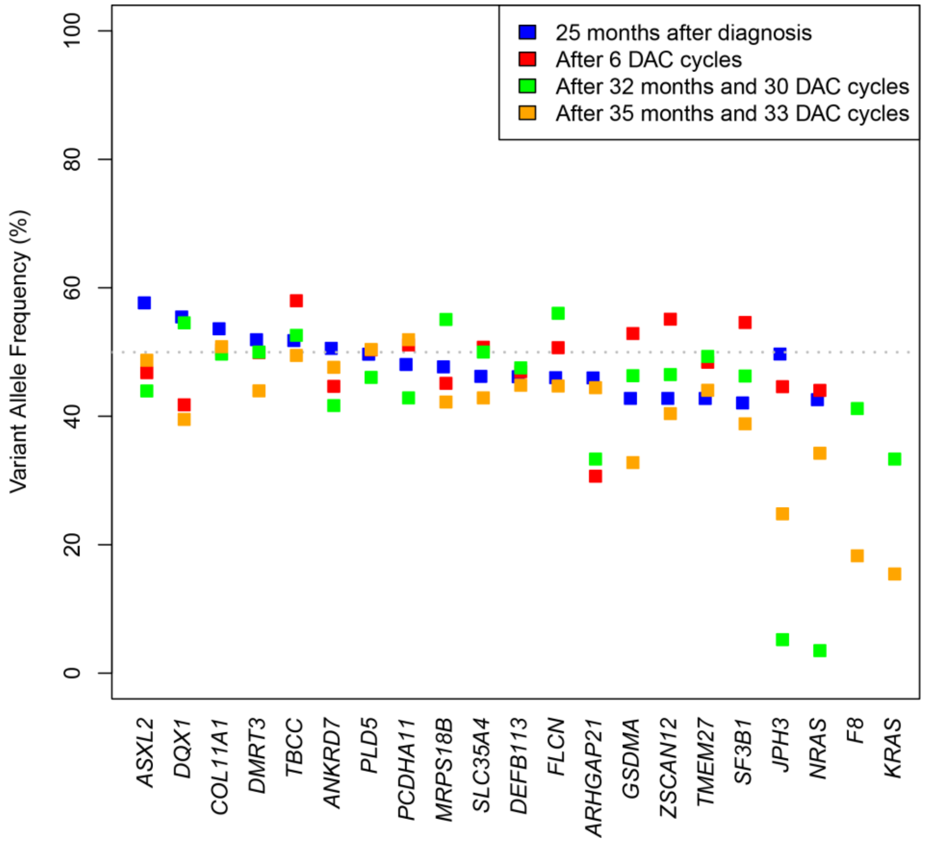

6. Serial whole exome sequencing in patient 35

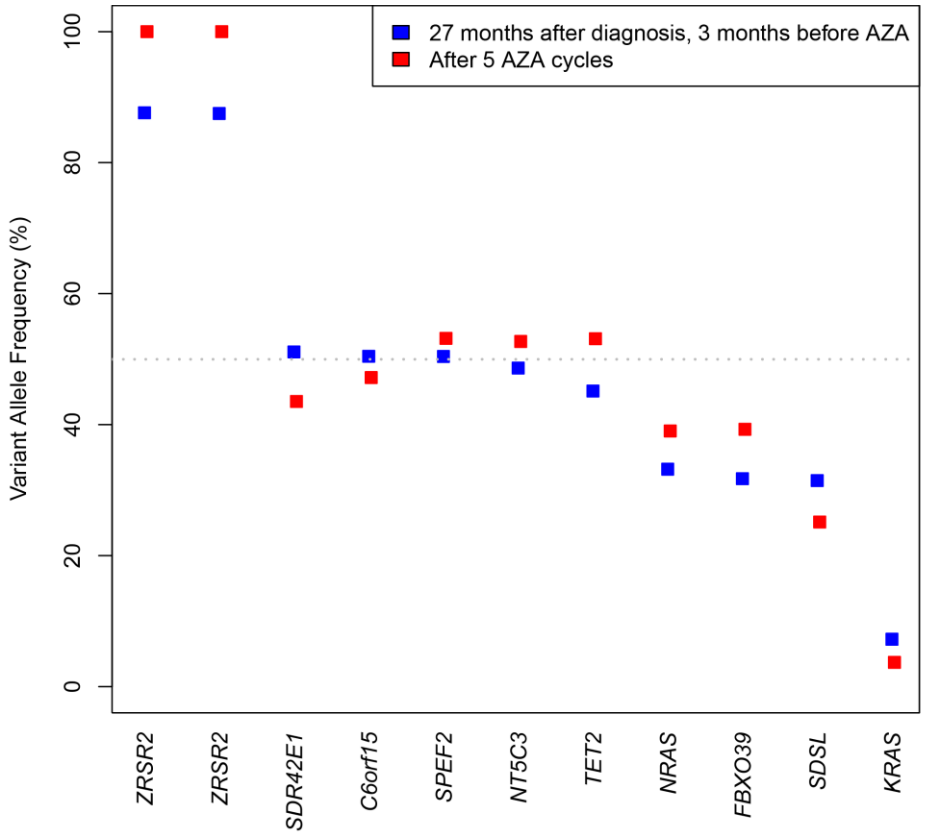

7. Serial whole exome sequencing in patient 48

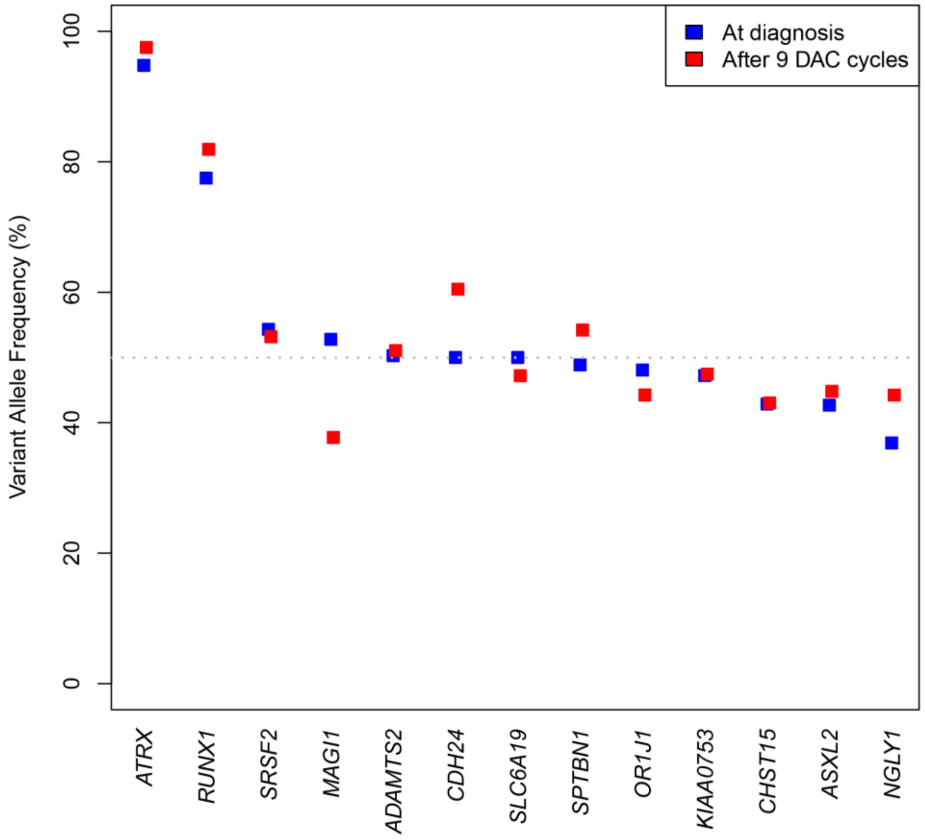

8. Serial whole exome sequencing in patient 3

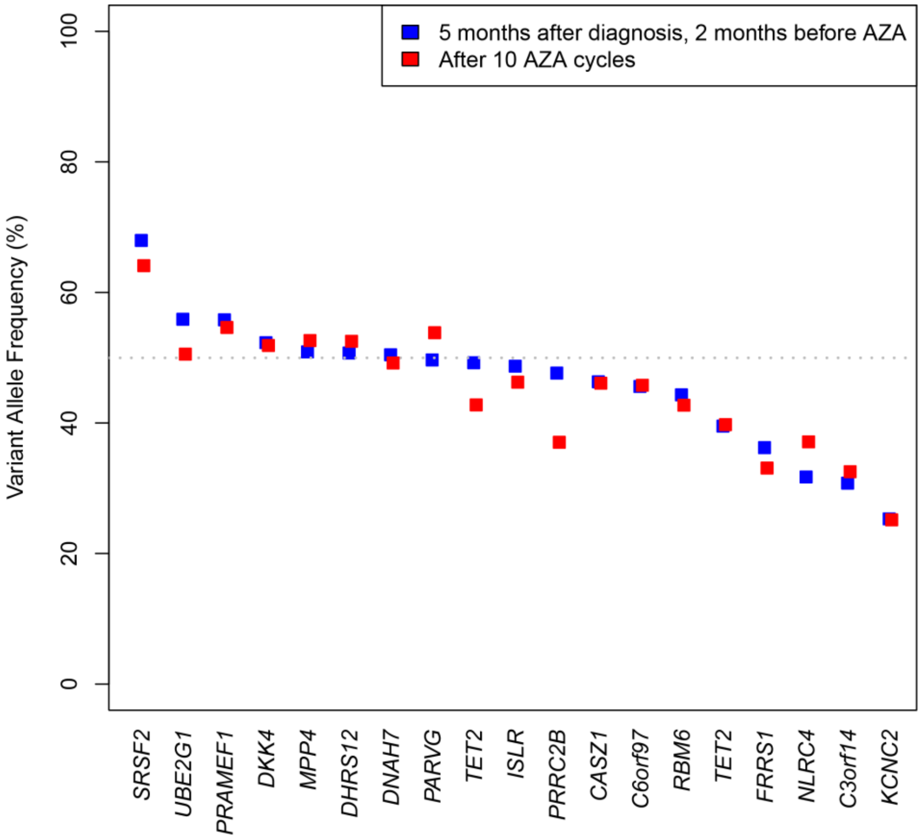

9. Serial whole exome sequencing in patient 32

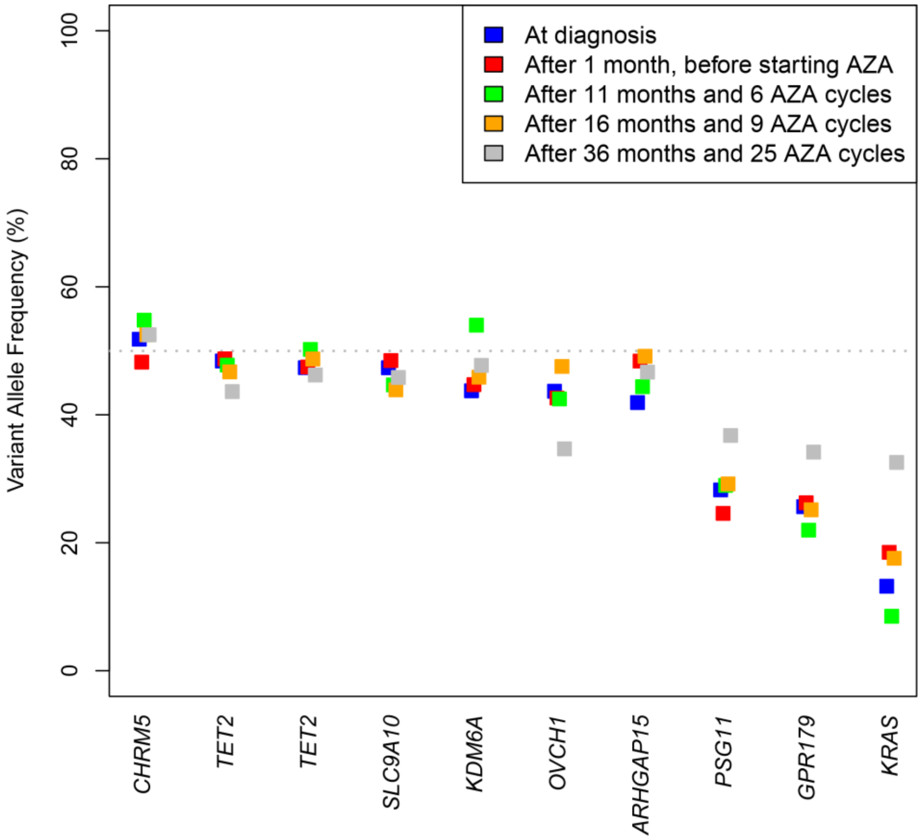

10. Serial whole exome sequencing in patient 1

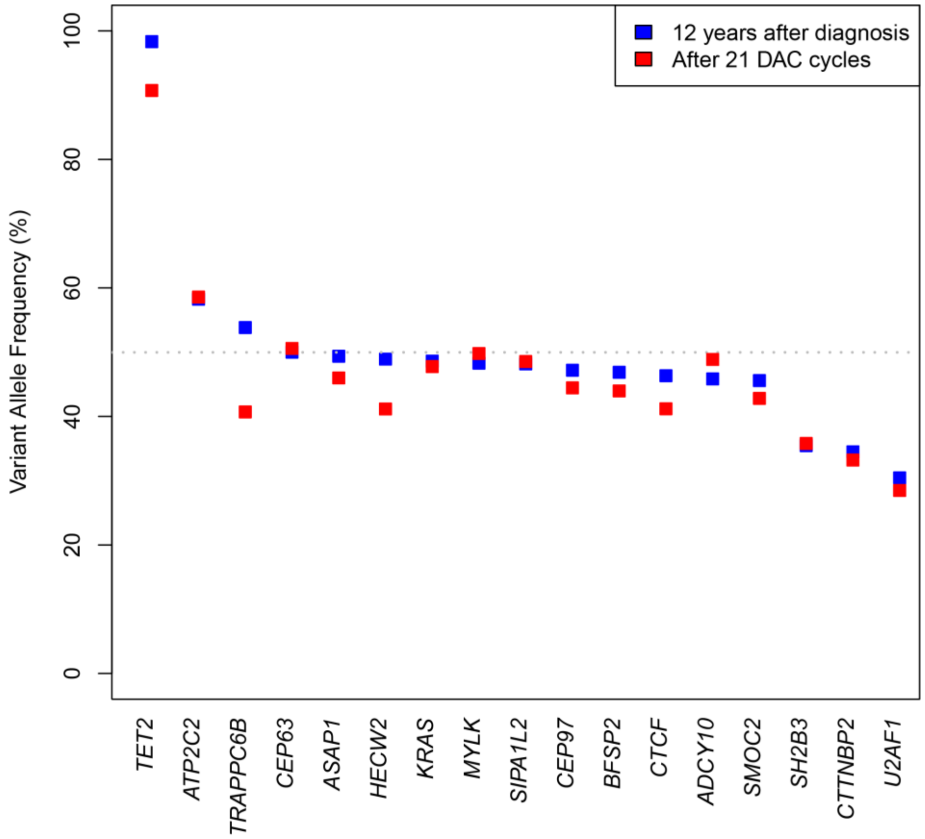

11. Serial whole exome sequencing in patient 21

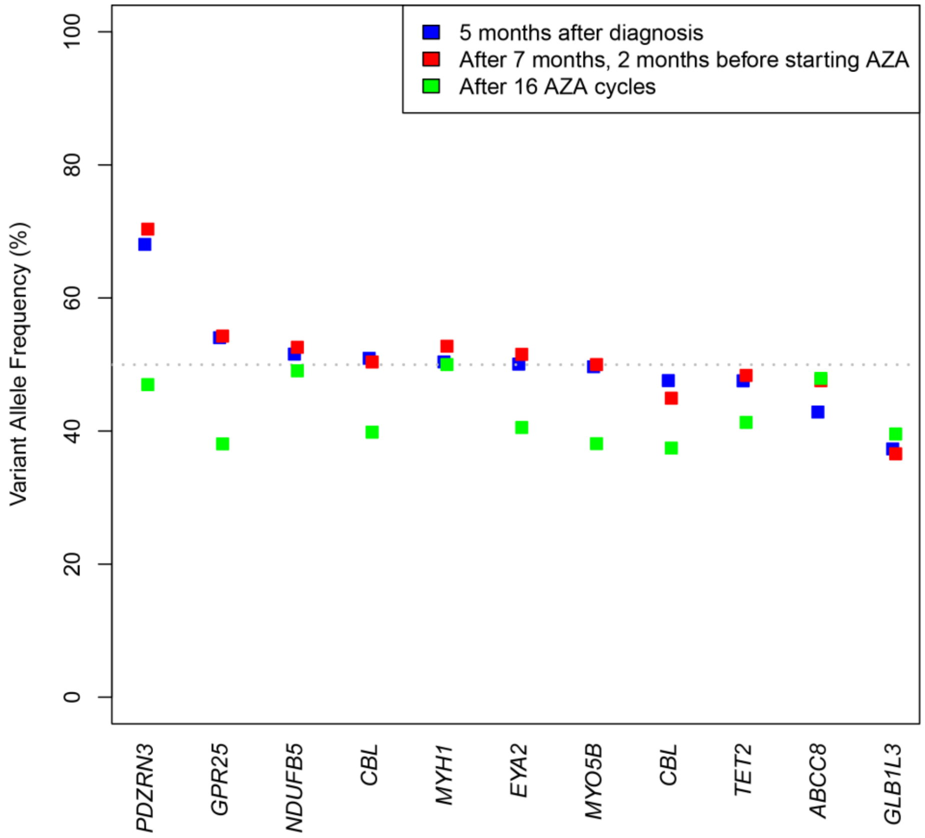

12. Serial whole exome sequencing in patient 5

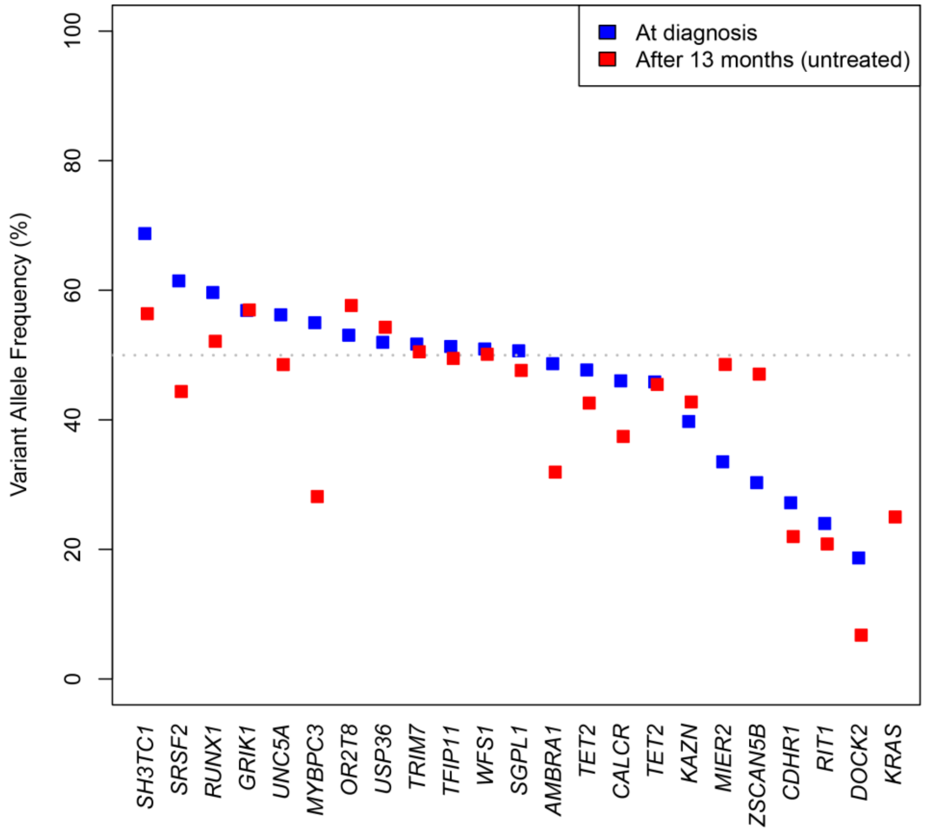

13. Serial whole exome sequencing in patient 9

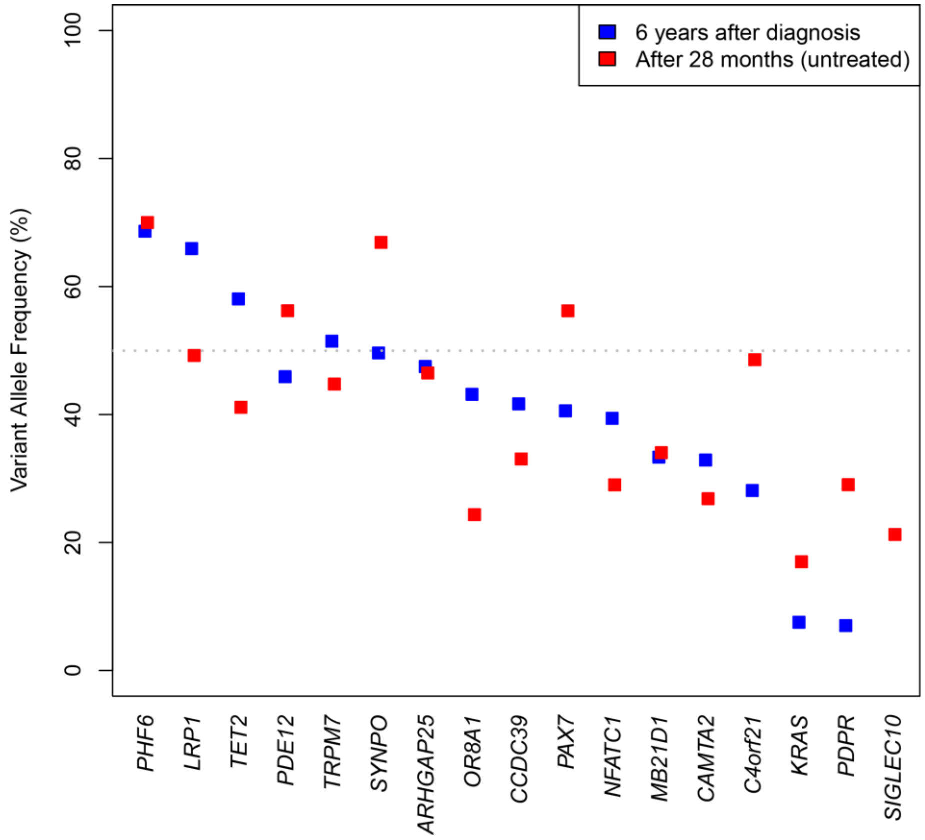

14. Serial whole exome sequencing in patient 46

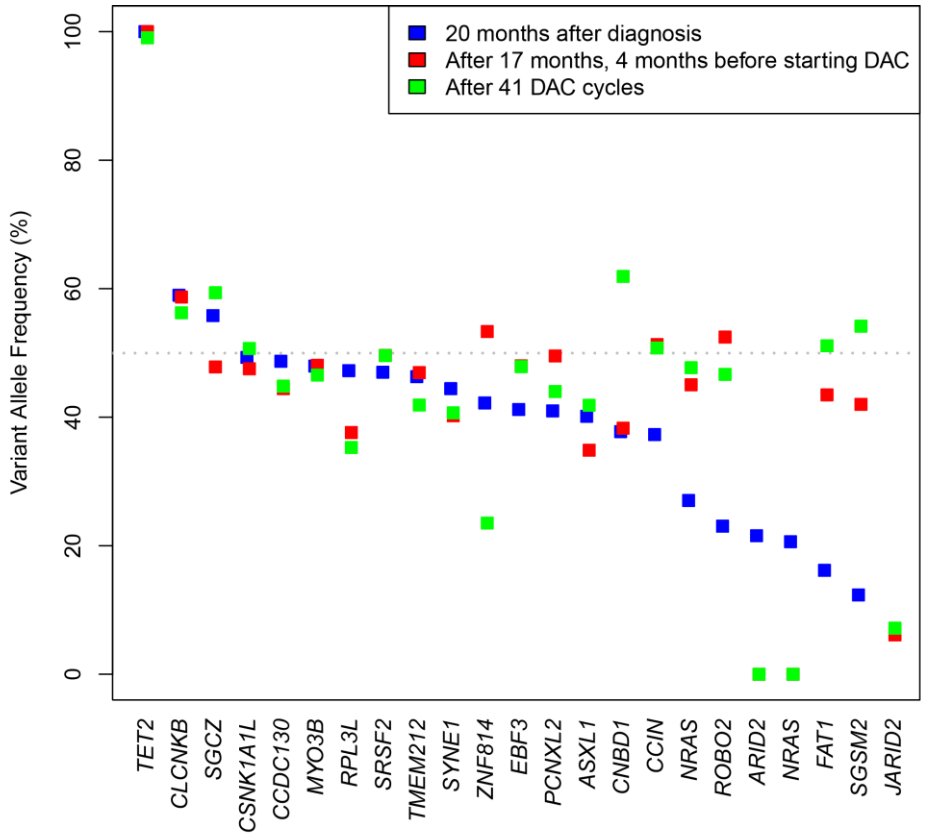

15. Serial whole exome sequencing in patient 49

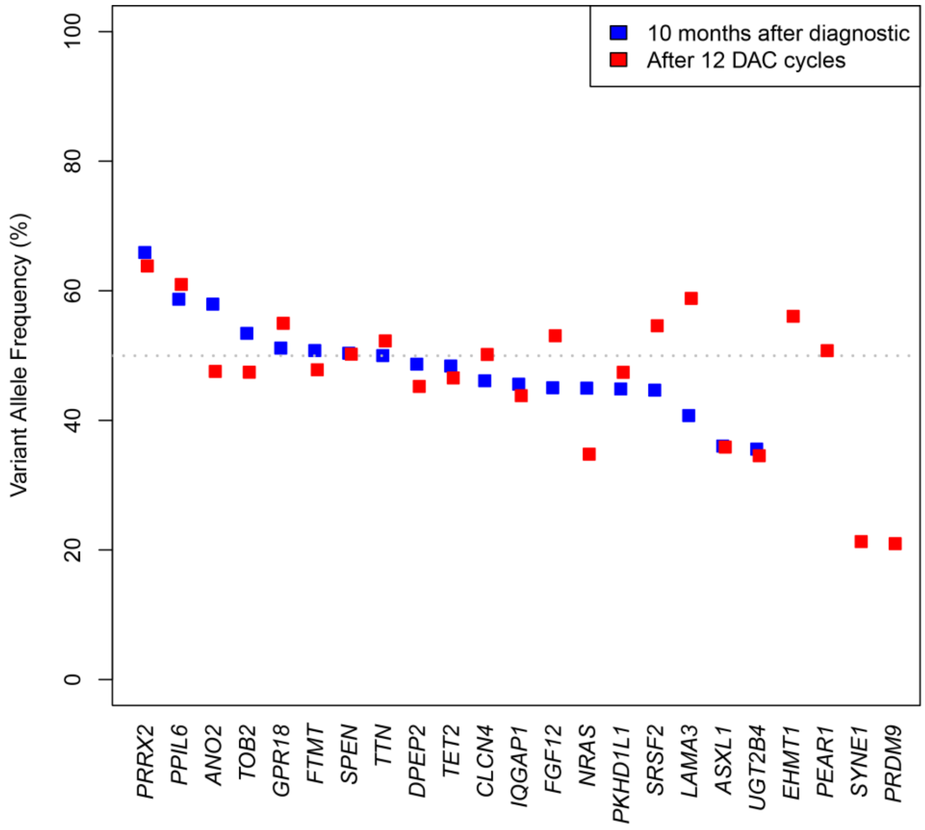

16. Serial whole exome sequencing in patient 28

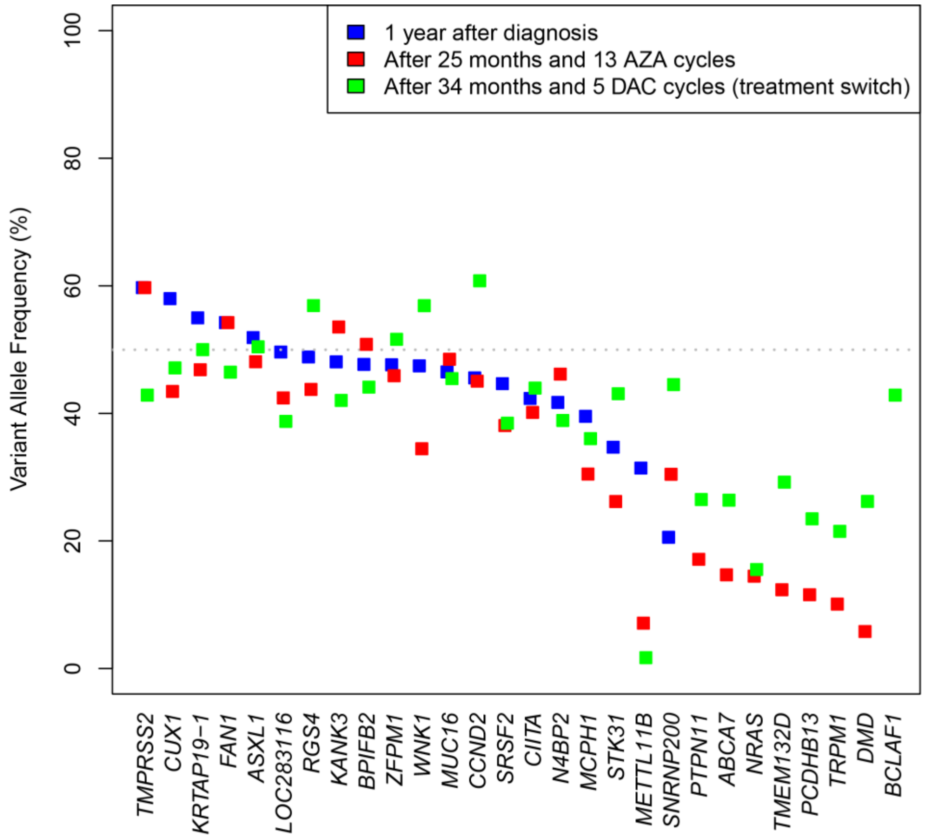

17. Serial whole exome sequencing in patient 34

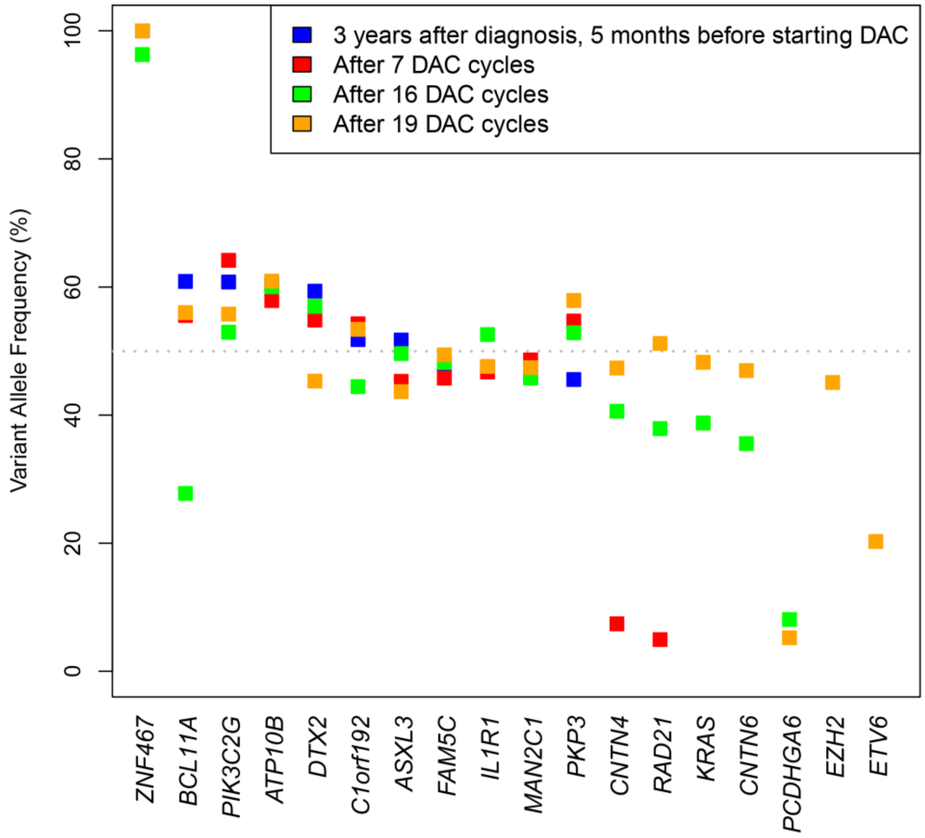

Supplementary Figure 6: Comparison of whole exome sequencing in sorted CD14+ and CD34+ cells for UPN46 after treatment

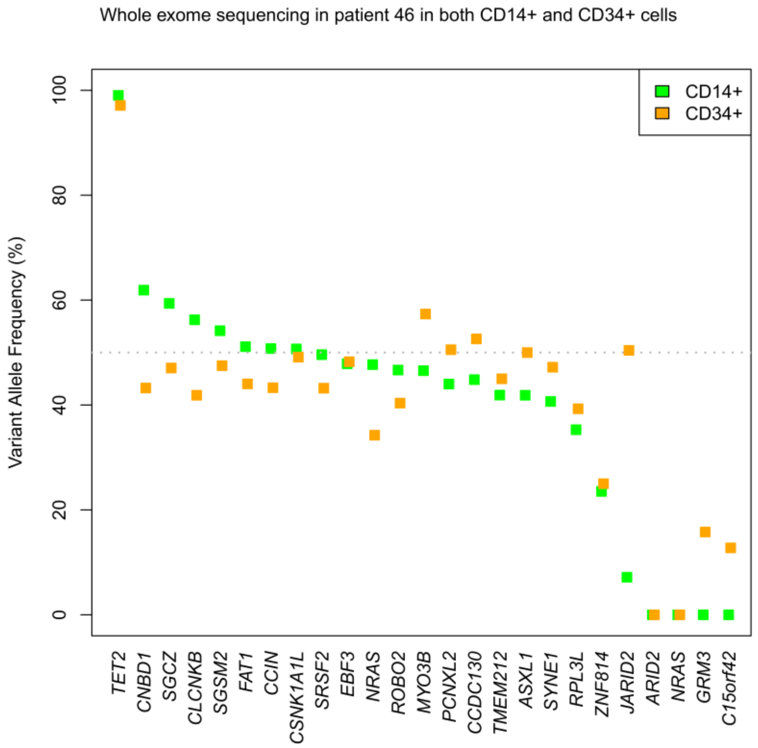

Supplementary Figure 7: RNA-Seq data validation. We compared indicated gene expression measured at two different time points in 9 patients by RNA-Seq and reverse transcription – quantitative polymerase chain reaction (RT-qPCR), respectively. For each studied gene, we measured gene expression induction at the second time-point compared to the first one. Correlations between the two methods are shown. RT-qPCR data were normalized to three independent reporter genes (*GUS*, *RPL32* and *HPRT*). Results plotted are those obtained with *GUS* normalization. R squared values generated by using RT-qPCR data obtained with each reporter are shown.

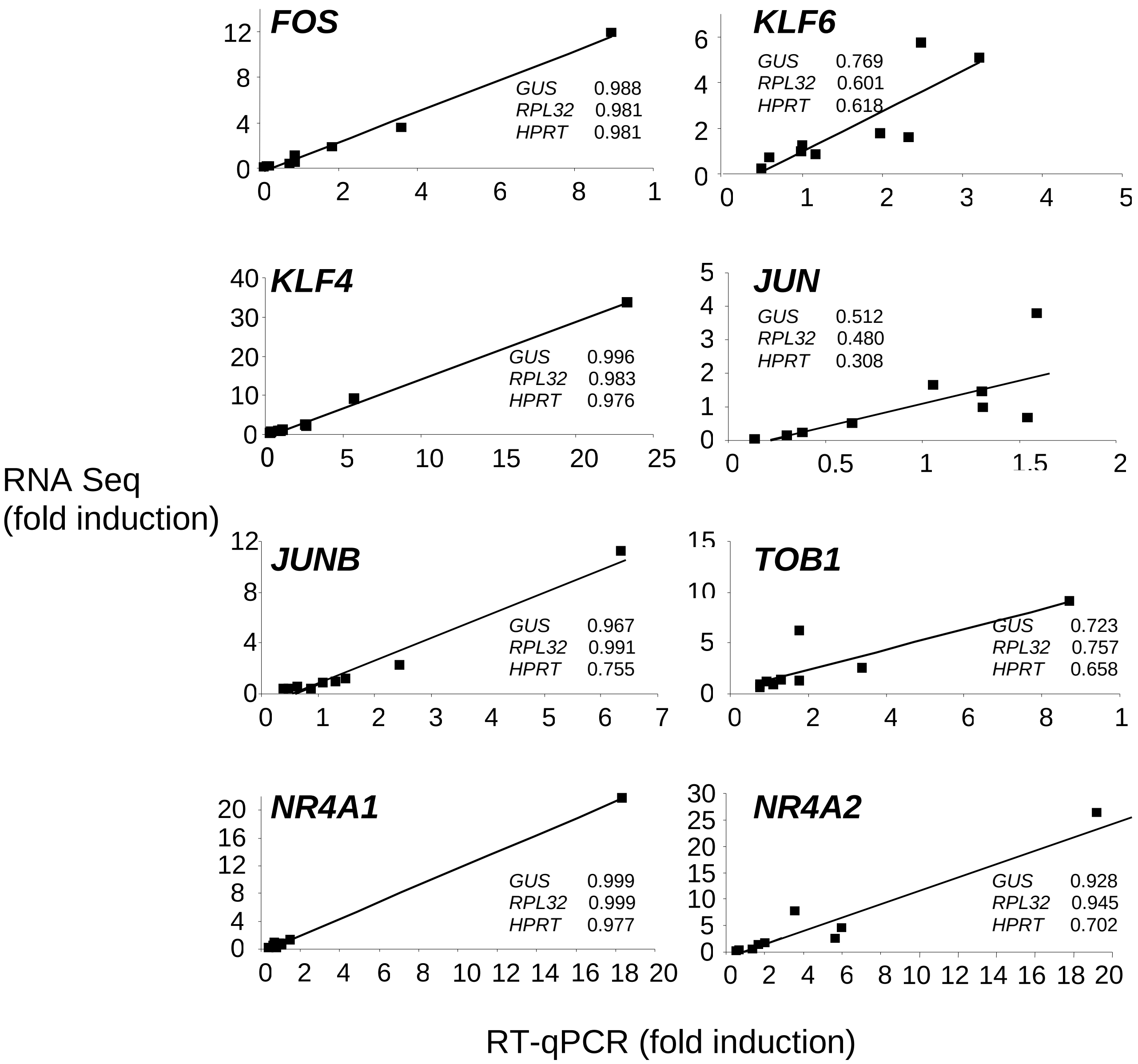

Supplementary Figure 8: Validation of RNA sequencing data. A and B: The differential expression of eight genes up-regulated in responders was explored in 6 responders (R, 3 studied by RNA-Seq in Figure 6b and 3 additional cases) and 10 non-responders (NR, 3 studied by RNA-Seq in Figure 6a and 7 additional cases). Data obtained with 2 housekeeping genes (*GUS* and *HPRT*) complement those obtained by using *RPL32* as normalizer. **C**: The differential expression of eight additional genes either up-or down-regulated in either responders or non-responders was explored the samples described above, using *RPL32* as normalizer. Again, genes detected as up-regulated in reponders were validated

A - *GUS* report

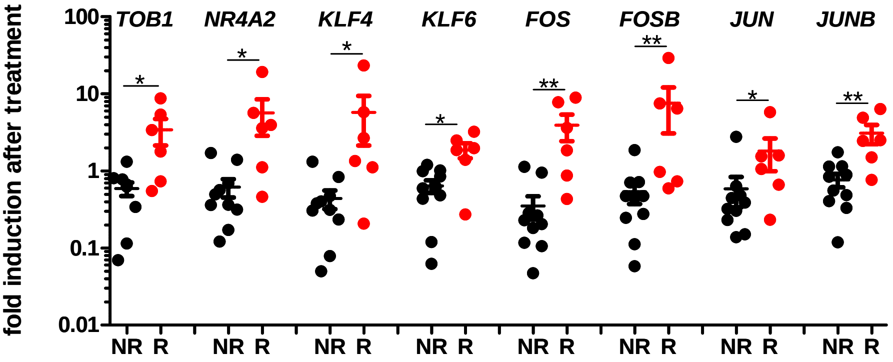

B – *HPRT* report

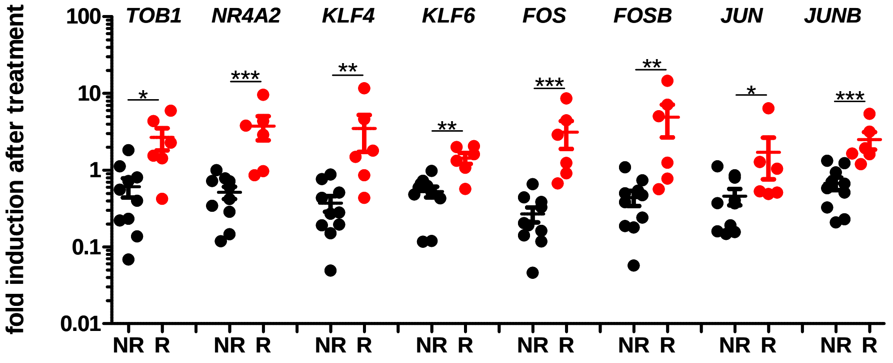

C - *L32* report

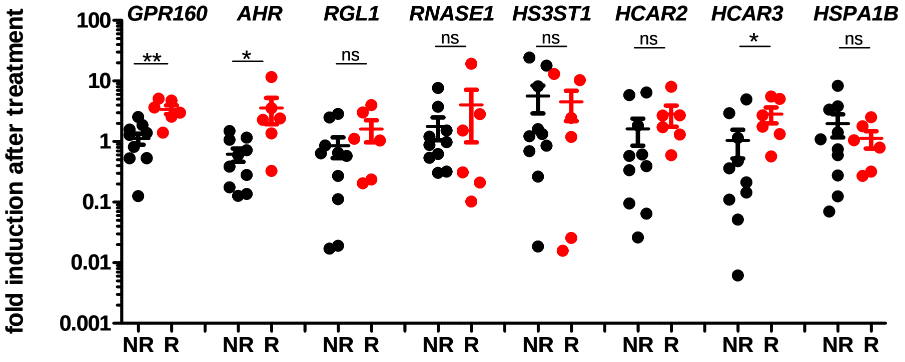

Supplementary Figure 9: Volcano plots and heatmaps of differentially methylated regions. These regions were studied twice in 9 patients, including 6 patients treated with a demethylating agents (responders: 3, non responders: 3) and 3 untreated patients.

A - Responders

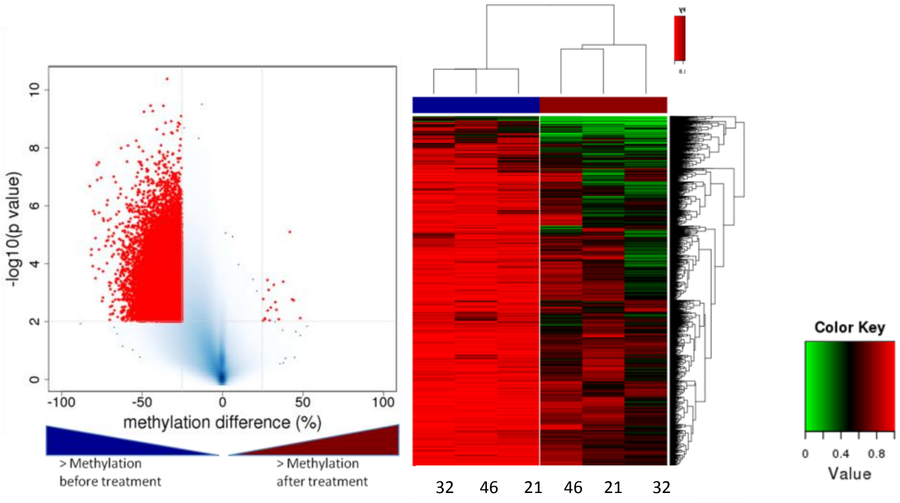

B – Non-responders (stable disease)

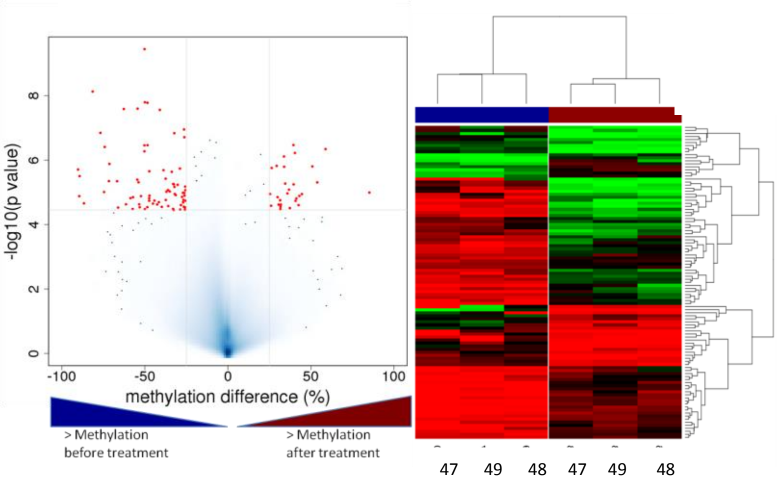

C - Untreated

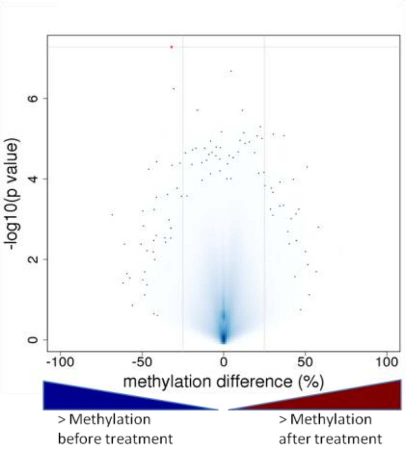

Supplementary Figure 10: Analysis of differentially methylated region (DMR) repartition. Pie charts illustrate the relative proportion of CpG tiles and DMRs annotated to the RefSeq promoter, exonic, intronic and intergenic regions, annotated to CpG islands, CpG shores and regions beyond CpG shores, and finally annotated to enhancers within gene bodies, intergenic and nonenhancer regions. **A**. In responders, differentially methylated regions were significantly depleted in promoters (5% vs. 24%, $p < 2.2\times10^{-16}$), as well as in CpGi (2% vs. 29%, $p < 2.2\times10^{-16}$), and significantly enriched in generic enhancers ($p < 2.2\times10^{-16}$). **B**. In non-responders (stable disease) remaining on therapy, DMRs, which were quantitatively much less important (see Figure 4), were also significantly depleted in promoters (4% vs. 27%, $p < 4.61\times10^{-10}$), as well as in CpGi (14% vs. 35%, $p < 1.14\times10^{-6}$), and hypo-DMRs were significantly enriched in generic enhancers compared with hyper-DMRs (21% vs. 11%, $p < 0.005$).

A - Responders

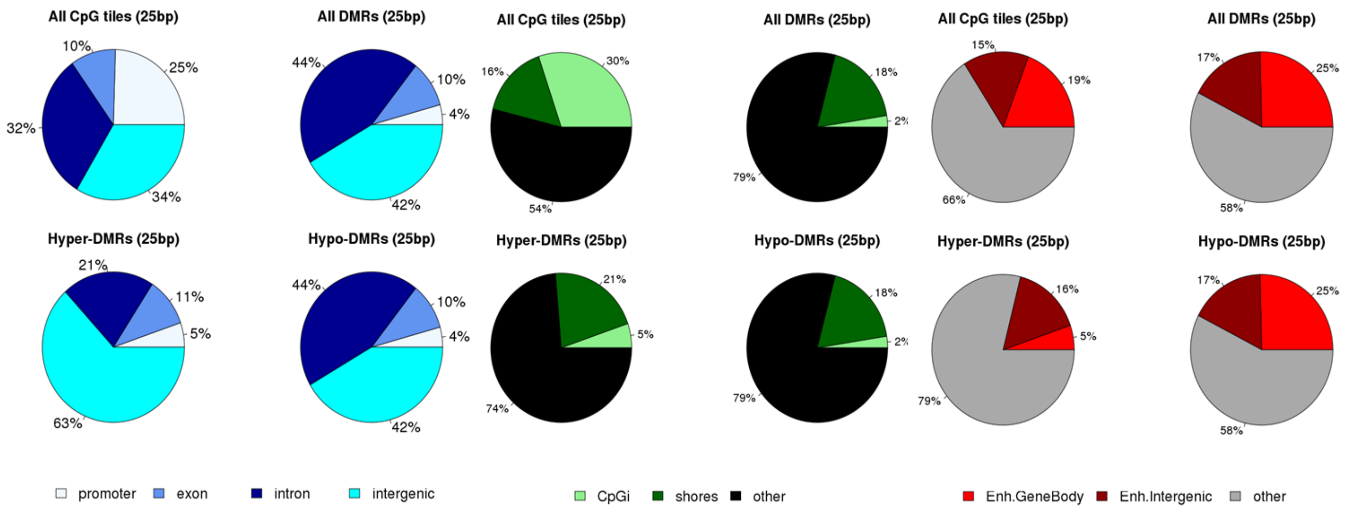

Non-responders (stable disease)

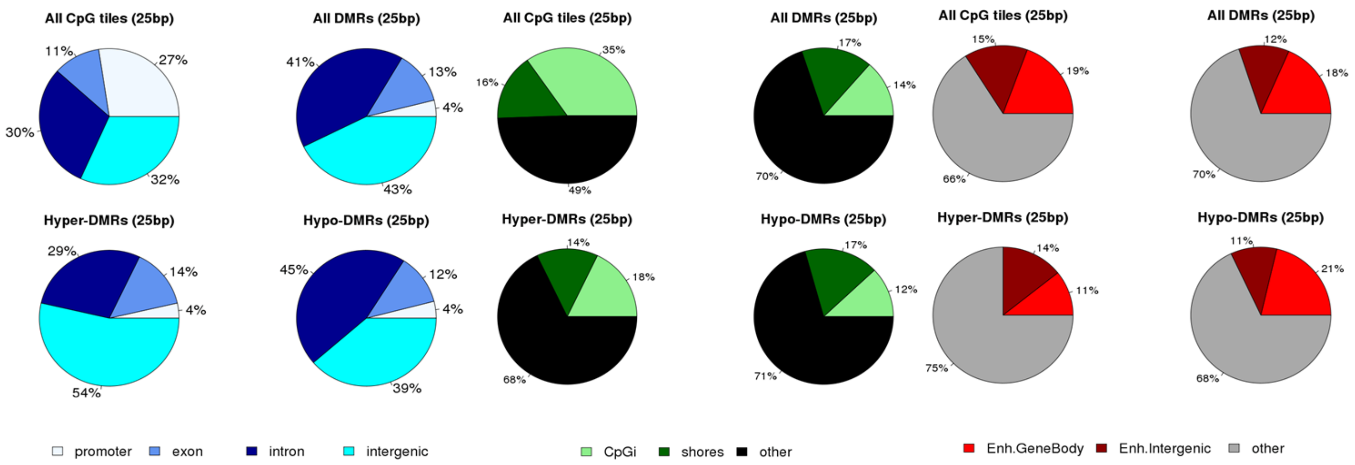

Supplementary Table 1: Characteristics of the three studied cohorts of patients.

	Whole exome (N=49)	Whole genome (N=17)	Validation (N=180)
**Clinical Information**			
Age in years : median (range)	74 (45-89)	71 (58-91)	74 (46-93)
Gender, n (%)			
Male	30 (61)	13 (76)	127 (71)
Female	19 (39)	4 (24)	53 (29)
Prior evolution, n (%)			
<6 months	28 (57)	8 (53)	80 (44)
>6 months	21 (43)	7 (47)	100 (56)
NA	0	2	0
WHO diagnosis			
CMML-1	37	15	158
CMML-2	12	1	22
NA	0	1	0
Cytogenetic risk*, n (%)			
Low	36 (74)	11 (65)	135 (75)
Intermediate	7 (14)	1 (6)	27 (15)
High	6 (12)	0	7 (4)
NA		5 (29)	11 (6)
WBC, 10^9/L, median (range)	14.7 (3.3-133,0)	8.8 (3.9-58)	10.3 (2.3-366.8)
Hemoglobin, g/dL, median (range)	11.8 (4.2-15.6)	12.2 (8.1-15.8)	11.3 (4.9-26.8)
Platelets, 10^9/L, median (range)	119 (6-1051)	104 (13-320)	117 (3-1427)
Monocytes, 10^9/L, median (range)	1.9 (1.0-62.8)	1.66 (1.2-17.8)	1.97 (1.0-84.4)
Peripheral Blasts %, median (range)	0 (0-14)	0 (0-6)	0 (0-9)
Bone Marrow Blasts %, median (range)	5 (0-17)	6 (2-18)	4 (0-18)
Immature myeloid cells,%, median (range)	0 (0-31)	0 (0-29)	0 (0-34)
Extramedullary-disease, n (%)			
Present	7 (15)	1 (6)	44 (24)
Absent	33 (67)	13 (76)	131 (73)
NA	9 (18)	3 (18)	5 (3)
**Mutational status (%)**			
TET2	59	65	64
SRSF2	47	18	44
ASXL1	33	12	32
CBL	20	12	16
KRAS	16	6	14
NRAS	16	12	13
DNMT3A	12	0	6
U2AF1	10	6	11
RUNX1	10	6	20
SF3B1	10	0	10
ZRSR2	8	6	11
CUX1	6	0	ND
EZH2	6	6	9
IDH2	6	0	12
LUC7L2	6	0	0
BCOR	6	0	0/68
JAK2	4	0	12
SH2B3	4	0	ND
ETNK1	4	0	3

NF1	6	0	6
ASXL2	4	0	0
DOCK2	4	0	2
ABCC9	4	0	2
HUWE1	4	0	1
TTN	4	0	ND
PHF6	4	0	8

# evolution : time from diagnosis to sampling
* According to the Spanish CMML cytogenetic classification. Low: normal and isolated -Y ; intermediate : other abnormalities and high : trisomy 8, complex caryotypes (>=3 abnormalities) and abnormalities of chromosome 7.
Immature myeloid cells include promyelocytes, myelocytes and metamyelocytes detected in the peripheral blood. NA, not available. ND, not done.

Supplementary Table 2: Whole exome sequencing and gene re-sequencing. Control samples were either sorted $CD3^+$ lymphocytes or skin fibroblasts or buccal swabs. T1, T2, T3, T4, T5 indicate the numbering of serial sequencing.

	Whole exome sequencing					
**Sample type**	**Control**	**Tumor - T1**	**Tumor - T2**	**Tumor - T3**	**Tumor - T4**	**Tumor -T5**
Nb of samples	N=49	N=49	N=17	N=6	N=3	N=1
Total reads (Mean)	135257990	129478657	142904800	105178277	98540375	125196156
%uniqMach (Mean)	94.85	94.74	94.29	93,67	94,4	96,365
Total bases (Mean)	9011607882	8928770091	9511603200	7973397798	7376183179	9724634877
%onTarget (Mean)	62.98	62.99	62.84	64,57	61,23	61,674
1x (Mean)	83.06	83.32	93.47	97,5	96,52	98,817
10x (Mean)	78.49	79.4	88.46	93,66	91,63	98,38
20x (Mean)	73.21	74.7	82.1	90,01	86,56	97,447
Coverage (Mean)	112.31	111.22	122.29	102,8	89	119
Coverage (SD)	47.88	40.46	71	58,85	45,43	NA
Coverage (Range)	20-317	24-231	10-259	30-201	37-121	NA
	**Gene re-sequencing**					
**Sample type**	**Control**	**Tumor - T1**	**Tumor - T2**	**Tumor - T3**	**Tumor - T4**	
20x (Mean)	93.07	92.59	91.88	90.7	91.41	
Coverage (Mean)	755.32	756.26	785.73	634	861.5	
Coverage (SD)	238.5	255.48	494.21	176.78	754.48	
Coverage (Range)	386-1460	376-1577	314-1984	509-759	328-1395	

NA: not applicable

Supplementary Table 3: Targeted re-sequencing of recently identified and previously unknown recurrently mutated genes. (*ASXL2*, *PHF6*, *DOCK2*, *NF1, ABCC9*, *HUWE1, ETNK1, LUC7L2*). Mutations were validated using MiSEq in 180 samples.

Sample type	Tumor
Nb of samples	N=180
20x (Mean)	92.02
Coverage (Mean)	690.11
Coverage (SD)	459.62
Coverage (Range)	96-2888

Supplementary Table 4: List of variants detected by targeted re-sequencing of previously unknown recurrently mutated genes.

Gene	Mutation type	RefSeq	Amino acid change	Nucleotide change	Mutated patients (training set N=49)	Mutated patients (validation set N=180)
PHF6	Nonsynonymous	NM_001015877	I314T	T941C	1	4
	FDel	NM_001015877	K26fs	76delA	0	1
	Stopgain	NM_001015877	E27X	G79T	0	1
	Stopgain	NM_001015877	L31X	T92A	0	1
	FDel	NM_001015877	G186fs	559delG	0	1
	Stopgain	NM_001015877	R225X	C673T	0	1
	FDel	NM_001015877	M243fs	729delG	0	1
	Nonsynonymous	NM_001015877	V268A	T803C	0	1
	Nonsynonymous	NM_001015877	R274Q	G821A	0	1
	Nonsynonymous	NM_001015877	G287D	G860A	1	0
	Nonsynonymous	NM_001015877	A288T	G862A	0	1
	Stopgain	NM_001015877	R319X	C955T	0	1
	Splice	NM_001015877		730-1G>T	0	1
	Splice	NM_001015877		1098+1G>A	0	1
NF1	FDel	NM_000267	260_260del	779_780delCC	0	1
	Nonsynonymous	NM_000267	V288M	G862A	0	1
	FInsert	NM_000267	*P370fs	1108_1109insCC	0	1
	FInsert	NM_000267	T676fs	2027_2028insC	0	1
	Nonsynonymous	NM_000267	L792H	T2375A	0	1
	Nonsynonymous	NM_000267	N793T	A2378C	0	1
	Nonsynonymous	NM_000267	R1276Q	G3827A	1	0
	Nonsynonymous	NM_000267	Y1587C		0	1
	Nonsynonymous	NM_000267	L1339R		0	1
	Stopgain	NM_000267	R1748X	C5242T	0	1
	Nonsynonymous	NM_000267	S1997N	G5990A	0	1
	Nonsynonymous	NM_000267	R2237Q	G6710A	0	1
	Splice	NM_000267		1185+1G>C	0	1
	Splice	NM_000267		A4760G	1	0
	Splice	NM_000267		204_205-2delAG	1	0
DOCK2	Nonsynonymous	NM_004946	M770V	A2308G	0	1
	Nonsynonymous	NM_004946	C853F	G2558T	0	1
	Nonsynonymous	NM_004946	R1189W	C3565T	1	0
	Nonsynonymous	NM_004946	L1208V	C3622G	1	0
	Splice	NM_004946		1258+5G>C	0	1
ABCC9	FDel	NM_005691	F472fs	1416delT	0	1
	Nonsynonymous	NM_005691	E607G	A1820G	1	0
	Nonsynonymous	NM_005691	T621I	C1862T	1	0
	FDel	NM_005691	*D1439fs	4317delT	0	1
	Splice	NM_005691	*	3566+1G>A	0	1
HUWE1	Nonsynonymous	NM_031407	R629H	G1886A	1	0
	Nonsynonymous	NM_031407	A4058V	C12173T	1	0
	Splice	NM_031407		2261+9T>G	0	1
ASXL2	Stopgain	NM_018263	R614X	C1840T	1	0
	FDel	NM_018263	E1172fs	3515delA	0	1

PHF6: 18 abnormalities were identified in 17 patients, including 14 distinct abnormalities and 2 variants in one of the patients (I314T and E27X); *NF1*: 15 abnormalities were identified in 14 patients, one patient carrying two variants, T676fs and L1339R; *ASXL2*: in addition to the shown variants, we detected 3 potentially germline SNVs (A497T, S185G, Q1371K) in 6, 3 and 2 patients respectively; smaller frequencies being reported in public databases; * indicates potential germline variants with an allelic frequency of 50 or 100% and no information in 1000G or ESP.

Supplementary Table 5: Correlations between mutated genes and clinical and biological parameters. We used Fisher exact test for qualitative parameters and Wilcoxon tests for quantitative parameters.

	**Positive correlation**	**Negative correlation**
**Hemoglobin level**	*TET2 (***)*	*ASXL1 (**), SF3B1 (**), ZRSR2 (**)*
**Platelet count**	*SF3B1 (***)*	*RUNX1 (***), SRSF2 (***)*
**White blood cell count**	*JAK2 (**), NRAS (***), ASXL1 (***)*	*PHF6 (***)*
**Monocyte count**	*NRAS (***), ASXL1 (**), SRSF2 (**)*	
**Peripheral blast cell count**	*ASXL1 (***), LUC7L2 (**)*	*TET2 (***)*
**Immature myeloid cell count**	*ASXL1 (***)*	
**Medullary blast percentage**	*KRAS (**)*	*JAK2 (***)*
**CMML2 WHO subgroup**	*KRAS (**)*	
**Low cytogenetic risk**		*TET2 (**)*

**: 0.001<P≤0.01 ; ***: P≤0.001

Supplementary Table 6: Alignment and coverage of whole genome sequencing

**Sample type**	**Control**	**Tumor**
Nb of samples	N=17	N=17
Total reads (Mean)	1028029614	1084281186
%uniqMach (Mean)	99.46	99.38
Total bases (Mean)	86842279017	91918801269
Nb covered bases (Mean)	2820703699	2819926295
Coverage (Mean)	30.18	32.12
Coverage (SD)	4.3	9.9
Coverage (Range)	27-44	25-59

Supplementary Table 7: Time between consecutive genomic analyses. The mean time between the two first time points in WES analyses was 12+/- 8 months in responders, 12 +/- 7 in non responders (stable disease), and 13 +/- 9 months in untreated patients.

UPN	Treatment Response	T1-T2 (months)	T2-T3 (months)	T3-T4 (months)	T4-T5 (months)	Mean (months)
1	Responder	21				21
3	Stable disease	12				12
5	Untreated	13				13
9	Untreated	28				28
21	Responder	7	18			12,5
23	Untreated	16				16
28	Stable disease	25,5	9			17,25
29	Untreated	4				4
30	Untreated	4,5				4,5
32	Responder	1	11	5	20	9,25
33	Untreated	15				15
34	Responder	12	9	3		8
35	Stable disease	8				8
46	Responder	17	47			32
47	Stable disease	6	26	3		11,7
48	Stable disease	9				9
49	Stable disease	12				12
Mean		**12,4**	**20**	**3,7**	**20**	**13,7**
SD		7,5	14,8	1,1	NA	7,5
Range		1-28	9-47	3-5	NA	4-32

Supplementary Table 8: Samples used for serial RNA-Seq and methylation experiments. AZA, azacytidine; DAC, Decitabine

UPN	Treatment	Status	RNA-Seq	ERRBS
**UPN5**	No		Yes	Yes
	No		Yes	Yes
**UPN23**	No		Yes	Yes
	No		Yes	Yes
**UPN9**	No		Yes	Yes
	No		Yes	Yes
**UPN21**	No		Yes	Yes
	AZA	Responder	Yes	Yes
**UPN32**	No		Yes	Yes
	AZA	Responder	Yes	Yes
**UPN46**	No		Yes	Yes
	DAC	Responder	Yes	Yes
**UPN47**	No		Yes	Yes
	DAC	Non Responder	Yes	Yes
**UPN48**	No		Yes	Yes
	DAC	Non Responder	Yes	Yes
**UPN49**	No		Yes	Yes
	DAC	Non Responder	Yes	Yes

Supplementary Table 9: RNA sequencing data alignment and coverage

Sample	RawR1/2	FilteredR1	FilteredR2	Left reads mapped	Right reads mapped	Over. Read alignment rate	Aligned pairs	concordant pair alignment rate	Reads On Transcriptome
UPN32_808	46808962	41294065	33925275	32364485 (78.4%)	30208145 (89.0%)	83.2%	28057834	79.7%	18060508
UPN32_1054	71231589	63156201	51852212	51366562 (81.3%)	47256823 (91.1%)	85.8%	44301062	82.2%	38728462
UPN46_227	120224282	117345119	110433131	109556388 (93.4%)	105364524 (95.4%)	94.4%	103453840	92.7%	88272219
UPN46_936	115308707	111565858	100280322	102577193 (91.9%)	94020431 (93.8%)	92.8%	92194563	90.7%	78459035
UPN21_969	122942488	119596084	108717931	110169667 (92.1%)	101511755 (93.4%)	92.7%	99702165	90.4%	88226310
UPN21_1284	52230173	48868690	32924089	40968346 (83.8%)	27634462 (83.9%)	83.9%	26937503	79.7%	34416112
UPN47_320	49482782	46217919	31099711	38350179 (83.0%)	25936814 (83.4%)	83.1%	25234168	79.0%	31235209
UPN47_408	108286314	104859767	94984982	95921983 (91.5%)	89302431 (94.0%)	92.7%	87370953	90.8%	76465420
UPN48_299	121708305	117891046	107449531	108434319 (92.0%)	101166905 (94.2%)	93.0%	99076424	90.9%	89400337
UPN48_426	38691148	32353829	16501191	22918394 (70.8%)	14644836 (88.8%)	76.9%	13074220	76.8%	2263141
UPN49_257	34995060	31286727	15008212	23192812 (74.1%)	13540416 (90.2%)	79.3%	12073219	75.1%	17626652
UPN49_433	118627675	116466600	108720777	106239580 (91.2%)	102765772 (94.5%)	92.8%	100519319	91.7%	83054517
UPN23_704	85241114	83736952	78455347	76106552 (90.9%)	73854796 (94.1%)	92.5%	72231454	91.4%	63159618
UPN23_939	133832338	130785724	120054128	116728703 (89.3%)	111186169 (92.6%)	90.9%	108613765	89.7%	93059490
UPN9_608	71956594	70176553	64136555	62891114 (89.6%)	59822073 (93.3%)	91.4%	58393627	90.2%	52557797
UPN9_1009	118307267	116598822	108180366	105229340 (90.2%)	102000602 (94.3%)	92.2%	99604907	91.5%	83850048
UPN5_732	45571594	44875674	42188768	40633204 (90.5%)	39893674 (94.6%)	92.5%	38963966	91.8%	33764802
UPN5_870	57207766	55328939	51138722	48906249 (88.4%)	47941566 (93.7%)	91.0%	46278280	89.4%	39243634
